# Eriodictyol ameliorates cognitive dysfunction in APP/PS1 mice by inhibiting ferroptosis via vitamin D receptor-mediated Nrf2 activation

**DOI:** 10.1186/s10020-022-00442-3

**Published:** 2022-01-29

**Authors:** Lin Li, Wen-Jun Li, Xiang-Ru Zheng, Qing-Long Liu, Qian Du, Yu-Jie Lai, Song-Qing Liu

**Affiliations:** 1 Department of Pharmacy, The Third Affiliated Hospital of Chongqing Medical University, Shuanghu Road, Yubei District, Chongqing, 401120 People’s Republic of China; 2 Department of Hepatobiliary and Pancreatic Surgery, The Third Affiliated Hospital of Chongqing Medical University, Chongqing, 401120 People’s Republic of China; 3 Department of Neurology, The Third Affiliated Hospital of Chongqing Medical University, Chongqing, 401120 People’s Republic of China

**Keywords:** Alzheimer’s disease, Eriodictyol, Ferroptosis, VDR, Nrf2

## Abstract

**Background:**

Alzheimer’s disease (AD) is the most common type of neurodegenerative disease in the contemporary era, and it is still clinically incurable. Eriodictyol, a natural flavonoid compound that is mainly present in citrus fruits and some Chinese herbal medicines, has been reported to exert anti-inflammatory, antioxidant, anticancer and neuroprotective effects. However, few studies have examined the anti-AD effect and molecular mechanism of eriodictyol.

**Methods:**

APP/PS1 mice were treated with eriodictyol and the cognitive function of mice was assessed using behavioral tests. The level of amyloid-β (Aβ) aggregation and hyperphosphorylation of Tau in the mouse brain were detected by preforming a histological analysis and Western blotting. HT-22 cells induced by amyloid-β peptide (1–42) (Aβ_1–42_) oligomers were treated with eriodictyol, after which cell viability was determined and the production of p-Tau was tested using Western blotting. Then, the characteristics of ferroptosis, including iron aggregation, lipid peroxidation and the expression of glutathione peroxidase type 4 (GPX4), were determined both in vivo and in vitro using Fe straining, Western blotting and qPCR assays. Additionally, the expression level of vitamin D receptor (VDR) and the nuclear factor erythroid 2-related factor 2/heme oxygenase-1 (Nrf2/HO-1) signaling pathway were tested using Western blotting and qPCR assays. Afterward, HT-22 cells with VDR knockout were used to explore the potential mechanisms, and the relationship between VDR and Nrf2 was further assessed by performing a coimmunoprecipitation assay and bioinformatics analysis.

**Results:**

Eriodictyol obviously ameliorated cognitive deficits in APP/PS1 mice, and suppressed Aβ aggregation and Tau phosphorylation in the brains of APP/PS1 mice. Moreover, eriodictyol inhibited Tau hyperphosphorylation and neurotoxicity in HT-22 cells induced by Aβ_1–42_ oligomer. Furthermore, eriodictyol exerted an antiferroptosis effect both in vivo and in vitro, and its mechanism may be associated with the activation of the Nrf2/HO-1 signaling pathway. Additionally, further experiments explained that the activation of Nrf2/HO-1 signaling pathway by eriodictyol treatment mediated by VDR.

**Conclusions:**

Eriodictyol alleviated memory impairment and AD-like pathological changes by activating the Nrf2/HO-1 signaling pathway through a mechanism mediated by VDR, which provides a new possibility for the treatment of AD.

**Supplementary Information:**

The online version contains supplementary material available at 10.1186/s10020-022-00442-3.

## Background

Alzheimer’s disease (AD) is the most common progressive neurodegenerative disease. According to the 2018 World Alzheimer Report, the number of patients with AD will increase to 152 million by 2050 as the population ages (Patterson 2018). Alzheimer’s disease has become a severe social problem. However, no ideal therapeutic drugs or treatments are currently available for AD.

Ferroptosis, which was first proposed by Dixon et al. in 2012, is a new form of non-apoptotic regulated cell death (RCD) that depends on the accumulation of intracellular iron and is characterized by the formation of lipid peroxides. Recently, an increasing number of findings have suggested that ferroptosis plays a vital role in the development of AD. One research group found that ferroptosis induced by a loss of ferroportin-1 (Fpn, also called SLC40A1) plays a critical role in the progression of AD. They explored whether genetic deletion of Fpn in principal neurons of the neocortex and hippocampus led to AD-like hippocampal atrophy, memory deficits and the canonical morphological and molecular characteristics of ferroptosis (Bao et al. 2021). Hambright et al. found that knockout of glutathione peroxidase type 4 (GPX4) in mouse cerebral cortex and hippocampal neurons induced degeneration of hippocampal neurons, resulting in obvious cognitive impairment. However, when mice were administered liproxstatin-1 (a ferroptosis inhibitor), the level of neurodegeneration was alleviated (Hambright et al. 2017). Additionally, a large cohort study found that ferroptosis increased the brain iron burden and the risk of accelerating the progression of AD (Ayton et al. 2021). Based on these findings, ferroptosis represents a promising research direction for developing anti-AD drugs.

**Fig. 1:**
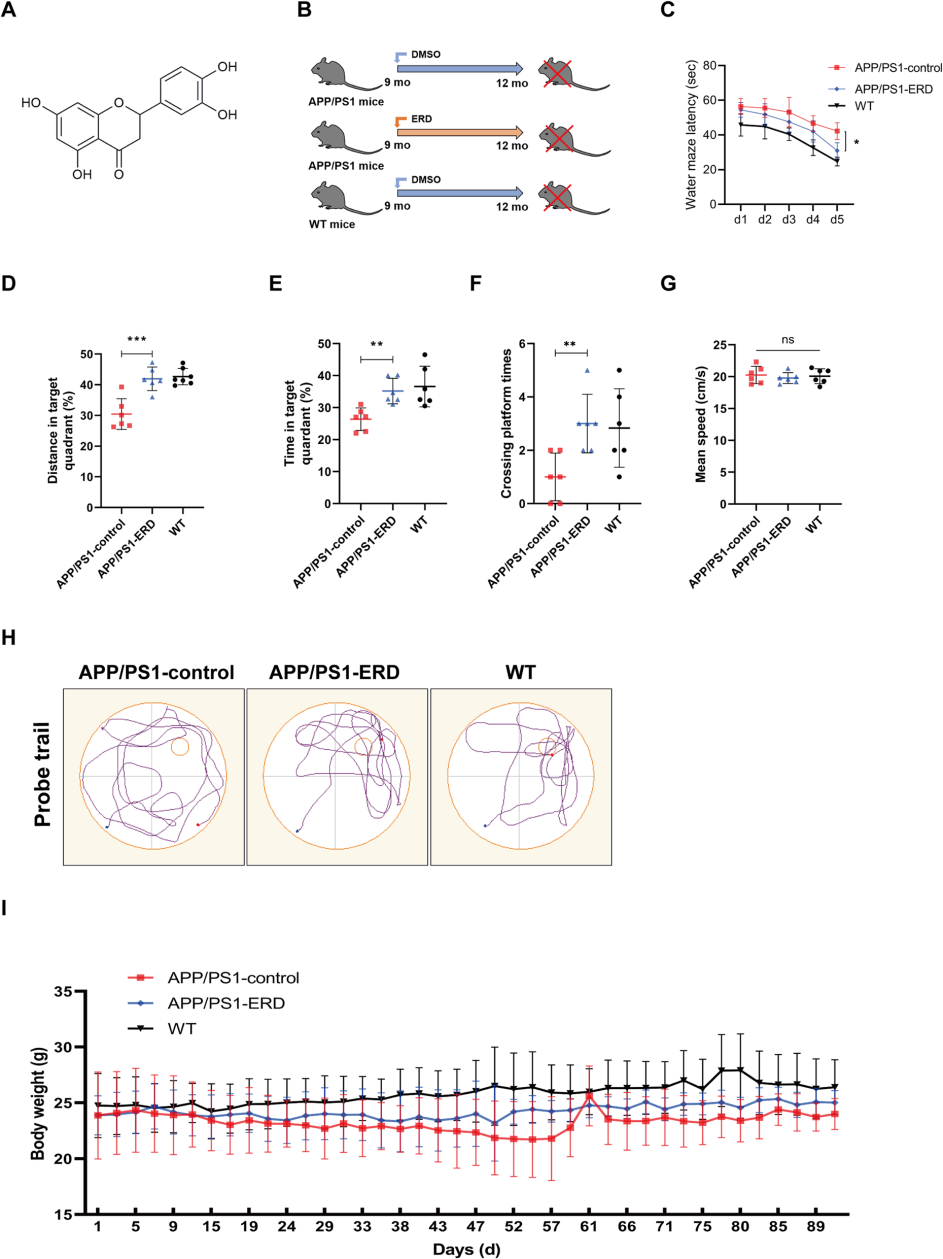
Eriodictyol ameliorates cognitive deficits in APP/PS1 mice, as measured using the Morris water maze. **A** The chemical structure of eriodictyol. **B** Animal grouping and experimental scheme for the effect of eriodictyol on memory deficits in APP/PS1 mice. **C** Escape latency of mice during the fifth day of platform trials. **D** The percentage of distance traveled in target quadrant. **E** The time spent in the target quadrant and **F** the number of platform crossings in the probe trials were determined. **G** The swimming speed of mice in the Morris water maze. **H** The track of the mouse in the Morris water maze was also recorded. **I** The body weight of each mouse was recorded during drug administration. The dose of eriodictyol was 50 mg/kg, and the mice were intraperitoneally injected three times a week for 3 months. Data are presented as means ± SD, n = 6. *P < 0.05, **P < 0.01 and ***P < 0.001 compared with the APP/PS1-control group

Recently, many natural products from plants or traditional medicines have shown great potential in AD prevention and treatment due to their multiple targets and low toxicity (Jiao et al. 2015; Wang et al. 2016; Pan et al. 2019). In particular, many flavonoids exert efficient neuroprotective effects and have become a hot research topic in preventing and treating Alzheimer’s disease (Yang et al. 2019; Uddin et al. 2020; Li et al. 2020a). Eriodictyol (ERD, the chemical structure is shown in Fig. 1A), a flavonoid compound, widely exists in the peel of citrus fruits and some Chinese herbal medicines (Lee et al. 2013). Existing studies have shown various biological activities of eriodictyol, such as anti-inflammatory, antioxidant and neuroprotective effects (Wang et al. 2017, 2020a; Bai et al. 2019; He et al. 2019). Jing et al. (2015) found that eriodictyol attenuates neuronal cell death induced by the Aβ_25–35_ peptide. According to another study, eriodictyol improves Lipopolysaccharide- (LPS-) induced cognitive impairments by inhibiting nuclear factor κB (NF-κB) in male C57BL/6J mice (He et al. 2018). Nevertheless, at present, the molecular mechanism underlying the anti-AD effect of eriodictyol has not been fully clarified.

In this study, we verified that eriodictyol exerts an anti-AD effect using APPswe/PS1E9 transgenic mice and the HT-22 cell line. In addition, eriodictyol inhibited ferroptosis in vitro and in vivo. Furthermore, we explored the underlying mechanism and found that eriodictyol activated the nuclear factor erythroid 2-related factor 2/heme oxygenase-1 (Nrf2/HO-1) signaling pathway through a mechanism mediated by vitamin D receptor (VDR). Thus, eriodictyol may be a potential drug for the treatment of AD and provides a promising regulatory target for flavonoid compounds in Alzheimer’s disease.

## Materials and methods

### Materials and regents

Eriodictyol (purity > 95%) was a gift from Syntech (SSPF) International, Inc (Montclair, CA). Aβ_1–42_ [amyloid β peptide (1–42), rat, J0426A] was purchased from Dalian Meilun Biotechnology, Co., Ltd. (Dalian, China). Iron Assay Kit, Congo Red Kit, Prussian blue, all-trans-retinoic acid (RA) and FITC-phalloidin were obtained from Sigma-Aldrich LLC. (Shanghai, China). Lipid Peroxidation MDA Assay Kit, Lipo8000, Propidium Iodide Test Kit (including DAPI), ROS Assay Kit and Calcein-acetoxymethyl (Calcein-AM) were purchased from Beyotime Biotechnology, Co., Ltd. (Shanghai, China). Protein A/G magnetic beads for Co-IP assay (B23201) were purchased from Bimake (Houston, TX, United States). The Cell Counting Kit-8 (CCK-8), All-in-One cDNA Synthesis SuperMix Kit and 2× SYBR Green qPCR Master Mix (Low ROX) were obtained from Bimake (Houston, TX, United States). The RNAsimple Total RNA Kit was purchased from TIANGEN Biotech Co., Ltd (Beijing, China). Anti-phospho-Tau (S396) (A5952), anti-ferritin (A5654), anti-GPX4 (A5569), and anti-lamin B1 (A5106) antibodies were also obtained from Bimake (Houston, TX, United States). Anti-SLC40A1 (26601-1-AP), anti-beta amyloid (25524-1-AP), and anti-Tau (66499-1-Ig) antibodies were purchased from ProteinTech Group, Inc. (Chicago, IL, United States). Anti-vitamin D receptor (AF6159), anti-Nrf2 (AF0639), anti-phospho-Nrf2 (Ser40) (DF7519), anti-HO-1 (AF5393), and anti-transferrin receptor (AF5343) antibodies were obtained from Affinity Biosciences (Zhenjiang, China). Goat anti-rabbit IgG H&L (IRDye 800CW) pre-adsorbed secondary antibody was obtained from Abcam (Cambridge, United Kingdom).

### Animals and treatments

APPswe/PS1E9 transgenic mice (APP/PS1 mice, female, 9 months old), a mouse model of Alzheimer’s disease, were obtained from Nanjing Junke Bioengineering Co., Ltd. (Nanjing, China) and wild-type C57 mice (female, 9 months old) were obtained from the Animal Experimental Center of Chongqing Medical University (Chongqing, China). All mice were bred in the Animal Experimental Center of Chongqing Medical University. All mouse husbandry procedures performed in this study were approved by the Chongqing Medical University Animal Welfare Committee. As shown in Fig. 1B, APP/PS1 transgenic mice were divided randomly into two groups (6 mice in per group): the model control group (saline, i.p., 3 times per week), and the ERD-treated group (50 mg/kg ERD, i.p., 3 times per week). Wild-type C57 mice were used as the positive control group (saline, i.p., 3 times per week). Mouse body weights were measured three times a week. After 3 months of continuous administration, behavioral tests were conducted on mice. Then, all mice were humanely euthanized and the brains were harvested immediately for further analysis (Jiao et al. 2015).

### Morris water maze test

The Morris water maze test consisted of four platform trials daily for 5 consecutive days, followed by a probe trial on the sixth day. Performance was video-recorded and analyzed using image analyzing software (ANY-maze, Stoelting). A circular pool was filled with water containing titanium dioxide at a temperature of 22–24 ℃, and a platform was placed in the first quadrant, approximately 1 cm below the water surface. In platform trials, the mice were placed in the water at one of the four quadrants. The time mice required from entering the water to find the platform and stand on it for 5 s was measured and recorded as the escape latency. Meanwhile, the swimming route was recorded. Notably, if the mice did not find the platform within 60 s, the escape latency was recorded as 60 s. In probe trials, the platform was removed, and the mice were allowed to swim in the pool for 60 s. Meanwhile, the swimming route, annulus crossings, swimming distance and time spent in each quadrant were recorded (Lai et al. 2019).

### Y-maze test

The Y-maze test included the spontaneous alternation test and the novel arm exploration test. The Y-maze consisted of three symmetrical arms at a 120° angle, and patterns of different colors were pasted on each arm to distinguish them (Lin et al. 2021). In the spontaneous alternation test, mice were allowed to move freely in the Y-maze for 8 min. The behavior of mice entering the three arms in succession on overlapping triplet sets was defined as a spontaneous alternation. The number of total entries and spontaneous alternations of mice were recorded. The percentage of alternation was calculated using the following formula: [the number of spontaneous alternations/(total number of arm entries − 2)] × 100% (Ding et al. 2020). In the novel arm exploration test, one arm was blocked by a baffle and defined as the novel arm, and mice were allowed to explore the other two arms (home arm and familiar arm) for 5 min. After a 2 h interval, baffle was removed and mice were allowed to freely explore all three arms for 5 min. The number of times the mice entered the novel arm, the distance traveled, and the time mice spent in the novel arm were recorded with a video camera. The Y-maze was cleaned with 75% ethanol after each trial.

### Congo red staining

As Aβ deposits in the brain can form plaques, the brain tissue samples were stained with Congo red (Rahman et al. 2019). The samples were fixed with 10% neutral buffered formalin. Next, dehydration and paraffin-embedding were performed and a microtome was used to prepare 5-μm tissue sections. The samples were stained with Congo red according to the manufacturer’s operating instructions. Then, the sections were observed and photographed at 400× and 100× magnification using a light microscope (Nikon, Japan).

### Fe staining

Perl’s Prussian blue staining was performed to intuitively observe the iron level in mouse brain tissues. Briefly, sections were incubated with a 1:1 mixture of 2% potassium ferrocyanide (Sigma-Aldrich, USA) and 2% hydrochloric acid for 10 min after deparaffinization and rehydration. Then, sections were rinsed with distilled water three times and the nuclei were counterstained with nuclear fast red (Beyotime Biotechnology, China). Finally, the sections were photographed under a fluorescence microscope at 400× and 100× magnification (Nikon, Japan) (Jahanshahi et al. 2021).

### Immunohistochemistry (IHE) analysis

Immunohistochemistry (IHE) was performed to detect Aβ, p396-Tau and VDR expression in the mouse brain tissues. Sections were deparaffinized and rehydrated before antigen unmasking using a target retrieval solution (Beyotime Biotechnology, China) in a boiling water bath for 10 min. After cooling to room temperature, endogenous peroxidase activity was quenched with a 0.3% hydrogen peroxide solution (H_2_O_2_) for 30 min. After washing, the sections were blocked with 1× TBST/5% normal goat serum for 1 h at room temperature and incubated with the following primary antibodies overnight at 4 ℃ in a humidified and light-protected chamber: anti-beta amyloid, anti-phospho-Tau (S396), anti-vitamin D receptor. Subsequently, a labeled polymer horseradish peroxidase anti-rat detection system was used according to the manufacturer’s instructions (Beyotime Biotechnology, China). Finally, the tissue sections were incubated with a 3, 3-diaminobenzidine (DAB) solution and observed with a light microscope (Nikon, Japan) at 400× and 100× magnification after during for 1–2 h (Streit et al. 2018).

### Cell culture and preparation of Aβ_1–42_

HT-22 hippocampal cells (HT-22 cells) were derived from HT-4 cells, which were immortalized from a primary mouse hippocampal neuronal culture. Cells were obtained from the Chinese Academy of Sciences (Shanghai, China) and cultured in Dulbecco’s modified Eagle’s medium (DMEM, Biological Industries, Israel) supplemented with 10% FBS (Biological Industries, Israel) and 1% penicillin/streptomycin (Beyotime Biotechnology, Shanghai). Cells were maintained in a sterile and humidified atmosphere at 37 ℃ with 5% CO_2_. Eriodictyol was dissolved in dimethyl sulfoxide (DMSO) and the final concentration of DMSO in culture medium was ≤ 1‰. The method for preparing Aβ_1–42_ oligomers was as follows: Aβ_1–42_ was dissolved in DMSO, sonicated for 5 min in a cold-water bath, and then immediately stored at – 80 ℃ as a stock. The Aβ_1–42_/DMSO solution was diluted with serum-free DMEM to a final concentration of 100 μM and stored at 37 ℃ until use (Sarlak et al. 2019).

### Cell viability assay

HT-22 cells were seeded in 96-well microtiter plates, and the cell density was approximately 3 × 10^3^ cells per well. The next day, the cells were incubated with fresh complete culture medium containing eriodictyol (0–128 μmol/L). After 48 h, the viability of HT-22 cells was detected using a CCK-8 assay according to the manufacturer’s instructions, and the absorbance was measured at 450 nm using a SynergyH1 microplate reader (BioTek). Similarly, the role of eriodictyol (0, 1, 2, 4, 8, 16 μM) in Aβ_1–42_ oligomer-induced cellular damage was measured using the CCK-8 assay, and the concentration of Aβ_1–42_ oligomers was 20 μM. When VDR was knocked out, the protective effect of ERD on HT-22 cells was still detected using the CCK-8 assay.

### Transmission electron microscopy (TEM) imaging

HT-22 cells were plated in 6 cm cell culture dishes and treated with or without Aβ_1–42_ oligomers and eriodictyol (2, 4, or 8 μM) for 48 h. HT-22 cells were collected and fixed with 2.5% glutaral for electron microscopy. The cellular ultrastructure was observed, and digital images were acquired using a JEM-1400 PLUS transmission electron microscope (TEM) (Hsieh et al. 2021).

### FITC-phalloidin assay

FITC-phalloidin was used to stain the cytoskeleton of HT-22 cells. HT-22 cells were plated in 24-well plates and cultured with culture medium containing 10 μM RA to induce neural axon formation for 3 days. Then, 20 μM Aβ_1–42_ oligomers were added to the culture medium after cells were pretreated with different concentrations of eriodictyol for 2 h, and culture medium was added to the vehicle control group. Forty-eight hours later, the cells were fixed with 4% (wt/vol) paraformaldehyde for 15 min and sequentially incubated with a FITC-phalloidin solution and DAPI at 37 °C. Finally, images were captured under a fluorescence microscope at 400× magnification and the length of axons was measured using ImageJ software (Jiao et al. 2015).

### Propidium iodide (PI) assay

Propidium iodide (PI) assay was performed to measure the cytotoxicity of Aβ_1–42_ oligomers and the protective effect of eriodictyol on HT-22 cells. HT-22 cells were seeded in 24-well plates at a cell density of approximately 1 × 10^4^ cells per well. The next day, HT-22 cells were pretreated with fresh complete culture medium containing different concentrations of eriodictyol or vehicle for 2 h and then incubated with Aβ_1–42_ oligomers at 37 ℃, while the control group was only treated with vehicle. After 48 h, the culture medium was removed and cells were washed with 1× PBS one time beforebeing stained with propidium iodide test kit obtained from Beyotime Biotechnology Company. Afterward, the cells were observed, and images were captured using a fluorescence microscope at 100× magnification (Huynh et al. 2021).

### Calcein-acetoxymethyl (Calcein-AM) assay

The level of iron in HT-22 cells was detected using calcein-AM. HT-22 cells were treated as described for the propidium iodide (PI) assay, and then the cells were incubated with calcein-AM for 30 min at 37 ℃. Photographs were captured with a fluorescence microscope (Prah et al. 2019).

### ROS assay

HT-22 cells were plated in 24-well plates and treated with 20 μM Aβ_1–42_ oligomers and different concentrations of eriodictyol on the second day. After 48 h, cells in every group were incubated with DHE or DCFH-DA (10 μM) for 20 min at 37 ℃ according to the manufacturer’s instructions for the ROS Assay Kit. Then, the cells were washed three times with serum-free cell culture medium. Cells were observe and images were captured using a fluorescence microscope at 100× magnification (Ge et al. 2021).

### Iron assay

The iron concentrations in the cortex, hippocampus and HT-22 cells were assessed according to the manufacturer’s instructions for Iron Assay Kit. Brain tissues (~ 10 mg) or HT-22 cells (2 × 10^6^) were rapidly homogenized in iron assay buffer and centrifuged to remove insoluble material. Then, samples (30 μL) were added to 96-well plates and incubated with 5 μL of iron assay buffer (ferrous iron) or iron reducer (total iron) at 25 ℃ to measure the ferrous iron or total iron content, respectively. After 30 min, 100 μL iron probe was added to each well and incubated for 60 min at 25 ℃ in a dark environment. Finally, the absorbance was measured using microplate reader at 593 nm.

### MDA assay

The malondialdehyde (MDA) content in the cortex, hippocampus and HT-22 cells was assessed using a Lipid Peroxidation MDA Assay Kit (Rahman et al. 2019). The test was performed according to the manufacturer’s instructions, and the absorbance was recorded with a microplate reader at 532 nm.

### Western blot analysis

Total protein was extracted from brain tissues or HT-22 cells. Nuclear and cytosolic proteins of HT-22 cells were extracted with a Nuclear and Cytoplasmic Protein Extraction Kit according to the manufacturer’s instructions (Yoo et al. 2017). The protein concentration was measured using an Enhanced BCA Protein Assay Kit (Beyotime Biotechnology, China). Proteins were diluted with 5× sodium salt/polyacrylamide gel electrophoresis (SDS/PAGE) sample loading buffer and denatured in a 100 ℃ metal bath for 10 min. Afterwards, protein samples were loaded on SDS/PAGE (8%, 10%, and 12%) gels, separated, and transferred to polyvinylidene fluoride (PVDF) membranes (Millipore, Billerica, MA, USA). Then, the blots were incubated with the following primary antibodies overnight at 4 ℃: anti-Tau, anti-phospho-Tau (S396), anti-beta amyloid, anti-xCT, anti-ferritin, anti-transferrin receptor, anti-Nrf2, anti-phospho-Nrf2 (Ser40), anti-HO-1, anti-GPX4, anti-vitamin D receptor, anti-anti-lamin B1 and anti-β-actin. Subsequently, the membranes were washed with 1× TBST three times and incubated with secondary antibodies for 2 h at room temperature. Next, the membranes were scanned using the Odyssey^®^ CLx Imaging System (LI-COR Biosciences, United States) (Prah et al. 2019; Li et al. 2020b). Finally, the gray values of the bands were compared using ImageJ software and GraphPad Prism 8.0.1 software.

### Real-time quantitative PCR (qPCR)

Total RNA was extracted from HT-22 cells using the Total RNA Extraction Kit according to the manufacturer’s instructions. Then, the total RNA was reverse transcribed into cDNA templates using the All-in-One cDNA Synthesis SuperMix kit according to the manufacturer’s protocol. Eventually, qPCR was performed with 2× SYBR Green qPCR Master Mix to measure mRNA expression. The gene-specific primers are listed in Table 1. The expression levels were normalized to the expression of β-actin and calculated with the 2^−ΔΔCt^ method (Orlandella et al. 2021).

**Table 1:** Primer sequences used for the qPCR analysis

Gene	Forward primer, 5ʹ-3ʹ	Reverse primer, 5ʹ-3ʹ
β-actin	GGCTGTATTCCCCTCCATCG	CCAGTTGGTAACAATGCCATGT
TfRC	GCCTTGCTCGGCAAGTAGAT	TCCTCCGTTTCAGCCAGTTT
FTH	AGAGCGGGCTGAATGCAATG	ATATTCTGCCATGCCAGCTTCAG
Fpn	CCAAGGCAAGAGATCAAACCCA	CAGGATGATTCCGCAGAGGAT
VDR	ATGAGGAGGTGCAGCGTAAG	CATCGAGCAGGATGGCGATA
GPX4	CCTTCCCCTGCAACCAGTTT	CCACGCAGCCGTTCTTATCA

### CRISPR/Cas9 system

CRISPR/Cas9 was used for VDR knockout (KO) (Jin et al. 2019). The single-guide RNA (sgRNA), sequence: 5ʹ-AGTCTGGAAAGCGTCACTTG-3ʹ, was cloned into lenti-CRISPRV2 plasmid and co-transfected into HEK-293 T cells with psPAX2 and pMD2.G and Lipo8000 to generate virus particles. Forty-eight hours after transfection, the virus particles in the cell culture fluid were filtered with a filter membrane (0.45 µm) and used to infected into the HT-22 cell line in the presence of polybrene (10 μg/mL) at 37 ℃. After 48 h, puromycin (2 μg/mL) was added to KO cells and NC cells to select stably infected cells. The efficiency of knockout was confirmed by Western blotting (Qu et al. 2019).

### Coimmunoprecipitation (Co-IP) assay

Total protein was extracted from of HT-22 cells treated with or without eriodictyol (8 μM) using IP lysis buffer (Beyotime, China), and the concentration was determined using an enhanced BCA protein assay kit. Then, a Co-IP analysis was performed with Protein A/G magnetic beads (Bimake, China, Shanghai) according to the manufacturer’s instructions (Yu et al. 2019; Wang et al. 2020b). Protein samples were diluted with binding buffer (50 mM Tris–HCl, 150 mM NaCl, and 0.1%–0.5 detergent (Triton X-100 and Tween 20), pH 7.5) to a final concentration of 100 μg/mL. Protein A/G magnetic beads were pretreated with binding buffer 3 times. Then, the Protein A/G magnetic beads were pre-incubated with the anti-Nrf2 or anti-vitamin D receptor antibody on a spinning wheel at room temperature for 15 min to allow the antibody to bind to the magnetic beads and washed 3 times with washing buffer (50 mM Tris–HCl, 150 mM NaCl, and 0.1%–0.5 detergent, pH 7.5). Afterward, the protein solution was added and co-incubated on a spinning wheel at room temperature for 1 h. After the protein solution was fully combined with the magnetic bead-antibody complex, the immunoprecipitate was washed three times with washing buffer and collected by separating magnetic beads. Then, the samples were resuspended in 1× protein loading buffer, heated for 10 min, and used for Western blotting assays with anti-vitamin D receptor or anti-Nrf2 antibody as the primary antibody. Subsequent the gray values of the bands in the resulting images were analyzed.

### Bioinformatics analysis

All microarray data were downloaded from the Gene Expression Omnibus (GEO) database (Accession no. GSE48350). The raw data were downloaded as MINiML files. The extracted data were normalized and processed using log2 transformation. The microarray data were normalized using the preprocessCore package in R software (version 3.4.1). Differentially expressed genes were identified using DESeq2 with the adjusted *P* value < 0.01. Correlations between gene expression were analyzed with the Spearman test.

### Statistical analysis

GraphPad Prism 8.0.1 software was adopted for statistical analyses. All data were analyzed by using two-tail Student’s t-test or one-way ANOVA with Tukey’s multiple comparison tests. *P* < 0.05 was considered statistically significant. All data are presented as the means ± SD, and the tests were repeated in at least three independent experiments.

## Results

### Eriodictyol ameliorates cognitive deficits in APP/PS1 mice.

First, we performed a Morris water maze experiment to test the spatial learning and memory functions of mice. On the fifth day of platform trials, the escape latency of APP/PS1 mice was longer than that of WT mice, while eriodictyol treatment significantly reduced the escape latency of APP/PS1 mice (Fig. 1C). Next, the platform was removed for probe trials. As shown in Fig. 1D–F, the swimming distance and the time spent in the target quadrant by APP/PS1 mice were shorter, and the number of platform crossings was fewer than that of WT mice. However, when APP/PS1 mice were intraperitoneally injected with eriodictyol, the proportion of distance traveled and the time spent in the target quadrant were substantially increased, and the number of platform crossings was increased. As shown in Fig. 1G, no difference in the mean swimming speeds of all groups of mice was observed, indicating that eriodictyol treatment did not affect the basic motor ability of APP/PS1 mice. The typical trajectories of each group of mice are shown in Fig. 1H. As expected, the results of the Morris water maze suggested that APP/PS1 mice showed significant spatial learning and memory deficits. However, eriodictyol treatment effectively improved these deficits in APP/PS1 mice and no significant difference in body weight of each group was observed during administration (Fig. 1I).

**Fig. 2:**
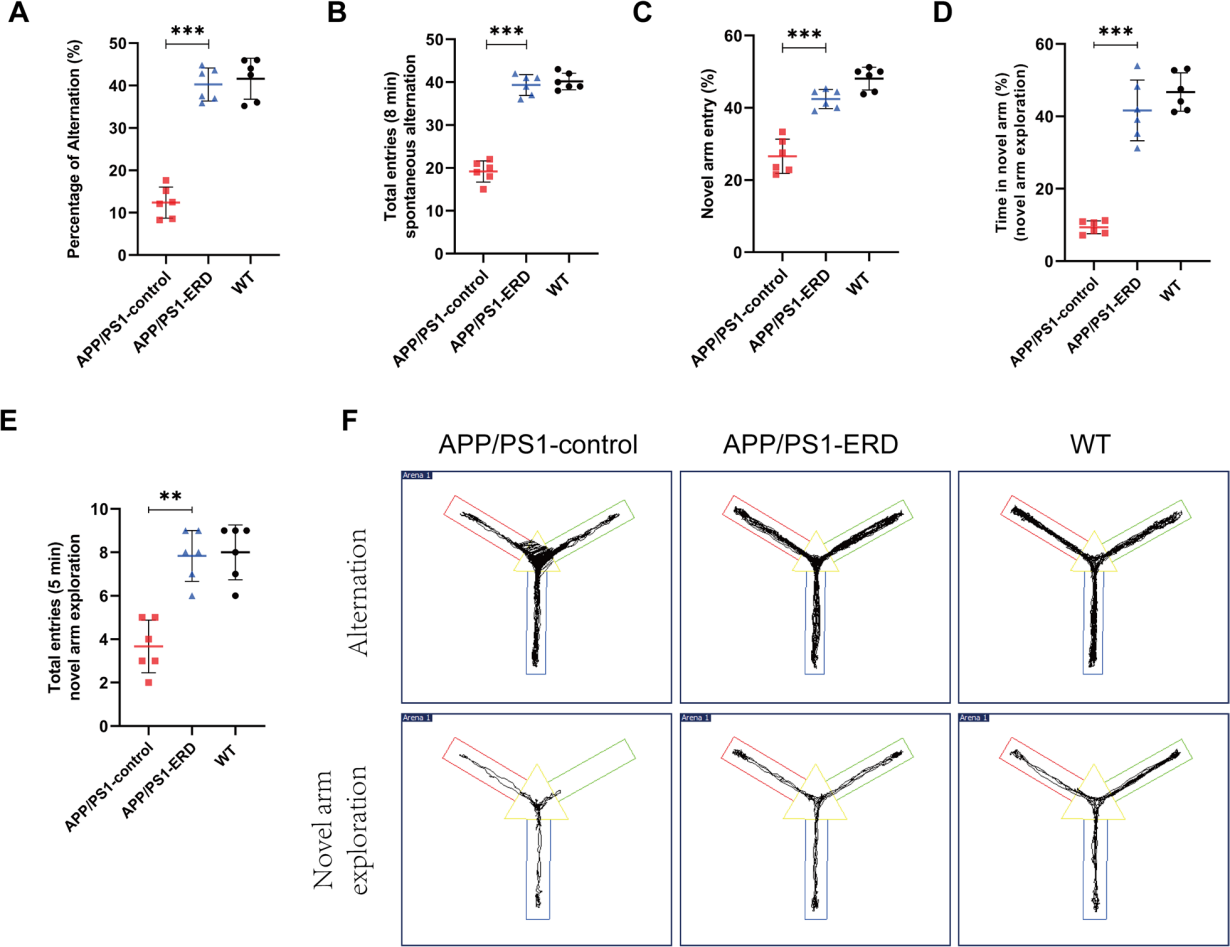
Eriodictyol ameliorates cognitive deficits in APP/PS1 mice determined using Y-maze tests. **A** Percentage of alternations and **B** total entries during 8 min were recorded in spontaneous alternation tests and novel arm exploration tests. **C** The percentage of novel arm entries. **D** The time spent in the novel arm and **E** the total entries within 5 min were recorded. **F** The track of mice in the Y-maze during spontaneous alternation tests and novel arm exploration tests. Data are presented as means ± SD, n = 6. **P < 0.01 and ***P < 0.001 compared with the APP/PS1-control group

Subsequently, Y-maze tests were conducted after the completion of Morris water maze tests to further investigate whether eriodictyol exerted a positive effect on the memory function of APP/PS1 mice. Consistent with the results of the Morris water maze tests, the results of Y-maze tests showed that the cognitive impairment in APP/PS1 mice was significantly ameliorated by eriodictyol treatment. Specifically, in spontaneous alternation tests, the percentage of spontaneous alternations and the number of total entries within 8 min were reduced in APP/PS1 mice compared with WT mice, while eriodictyol markedly reversed these changes (Fig. 2A, B). Then in novel arm exploration tests, we also found that the APP/PS1 mice treated with eriodictyol showed a higher percentage of novel arm entries, more time spent in the novel arm and a greater number of total entries within 5 min compared with the mice in the APP/PS1-control group (Fig. 2C–E). The representative track of each group of mice is shown in Fig. 2F. These results of behavioral tests indicated that eriodictyol ameliorates cognitive deficits in aged APP/PS1 mice.

### Eriodictyol alleviates Aβ aggregation and Tau hyperphosphorylation in APP/PS1 mice

**Fig. 3:**
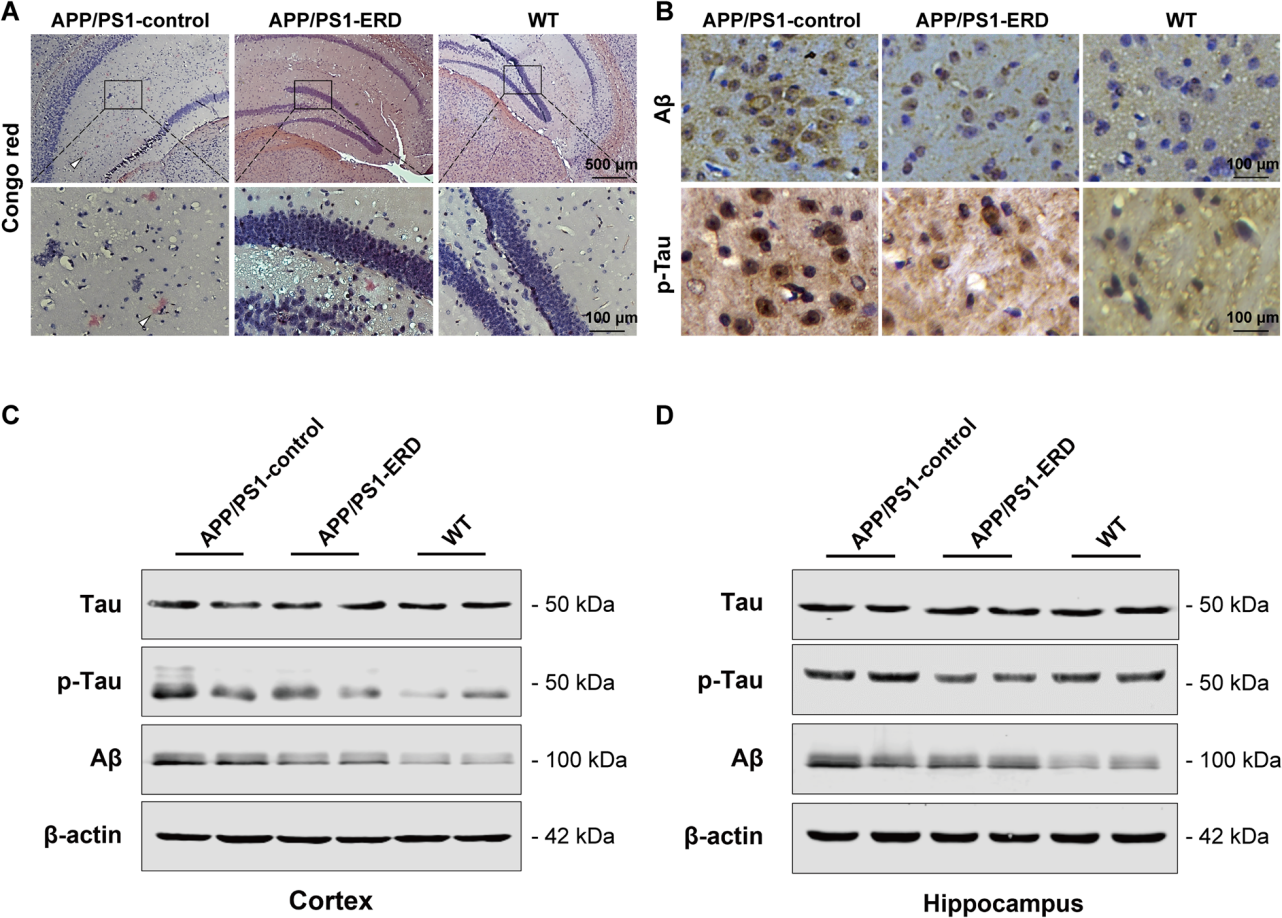
Eriodictyol alleviates Aβ aggregation and Tau hyperphosphorylation in APP/PS1 mice. **A** Aβ aggregation was measured using Congo red staining. **B** The levels of Aβ and p-Tau in the brains of mice were detected using IHE assay. **C**, **D** Levels of p-Tau, Tau and Aβ in the cortex and hippocampus of mice were measured using Western blot analysis. β-Actin was used as a loading control. The dose of eriodictyol was 50 mg/kg, and the mice were intraperitoneally injected three times a week for 3 months

We performed Congo red staining of amyloid plaques and IHE analysis of the Aβ protein and phosphorylated-Tau (p-Tau) levels to investigate whether eriodictyol exerted a beneficial effect on Aβ aggregation and Tau hyperphosphorylation in APP/PS1 mice. Images of Congo red staining are shown in Fig. 3A. We observed that eriodictyol markedly reduced Aβ aggregation in the brains of APP/PS1 mice. Similarly, the IHE assay revealed a marked reduction in levels of the Aβ protein in the brains of eriodictyol-treated APP/PS1 mice, and the level of p-Tau was also reduced by eriodictyol treatment (Fig. 3B). The results of Western blot assay were consistent with these findings, as the levels of Aβ and p-Tau in the cortex and hippocampus were reduced by eriodictyol compared with APP/PS1 control mice, despite the lack of a remarkable change in Tau levels (Fig. 3C, D and Additional file 1: Fig. S1A, B). These findings revealed that eriodictyol treatment alleviates Aβ aggregation and Tau hyperphosphorylation in APP/PS1 mice.

### Eriodictyol inhibits ferroptosis in the brains of APP/PS1 mice

**Fig. 4:**
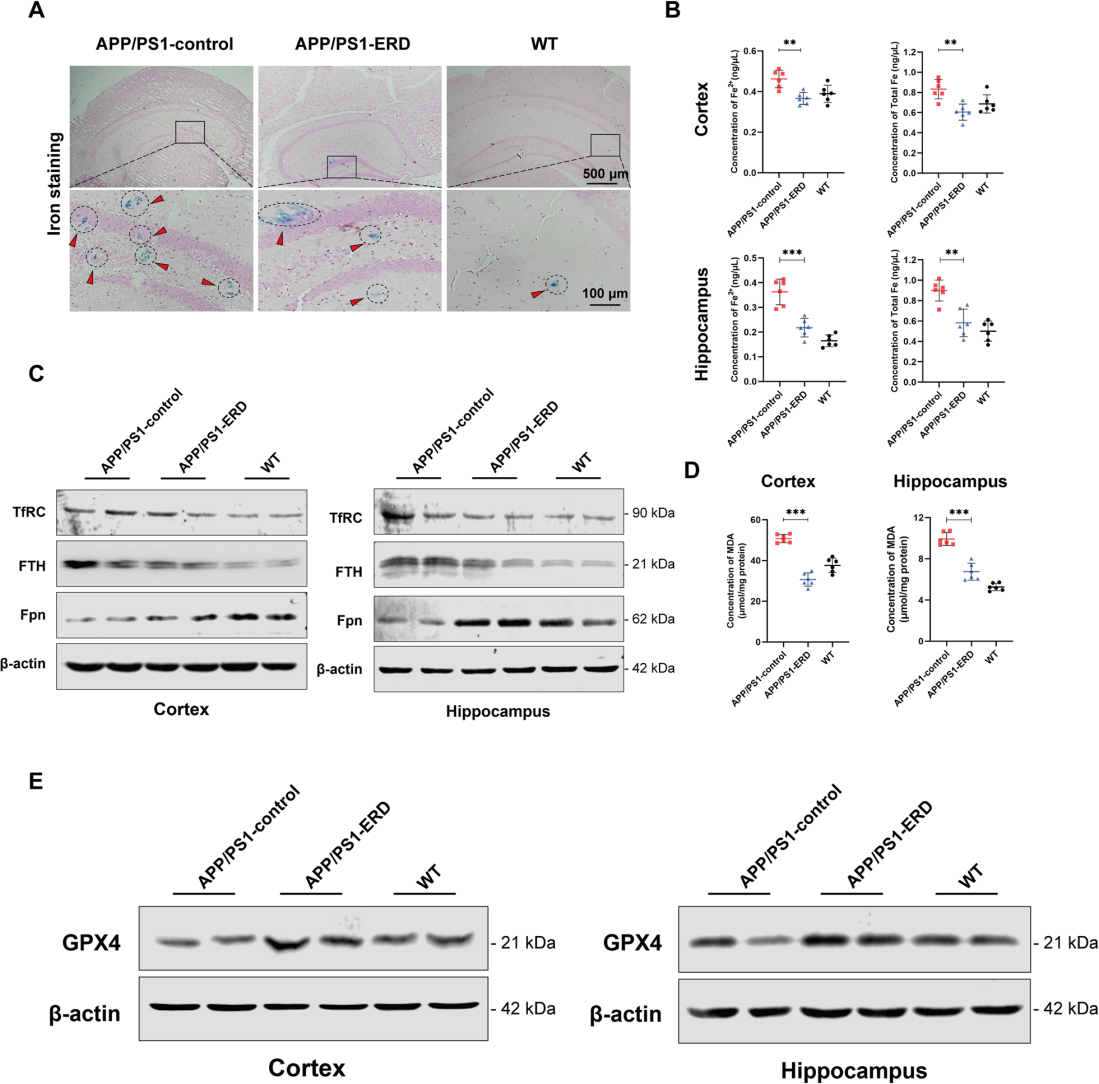
Eriodictyol inhibits ferroptosis in the brains of APP/PS1 mice. **A** Iron accumulation in the brains of mice was stained with Prussian blue. **B** The levels of ferrous iron and total iron in the mouse cortex and hippocampus were detected using an iron assay kit. **C** The expression of TfRC, FTH and Fpn in the mouse cortex and hippocampus was measured using Western blotting, and β-actin served as a loading control. **D** MDA contents in the mouse cortex and hippocampus were detected using a Lipid Peroxidation MDA Assay Kit. **E** The GPX4 expression level in the mouse cortex and hippocampus was measured using Western blotting. The data are presented as the means ± SD of three experiments. **P < 0.01 and ***P < 0.001 compared with the control group

Iron accumulation in the mouse brains was measured using Prussian blue staining. As expected, more iron deposits that were observed as blue spots after Prussian blue staining were observed in the brains of APP/PS1 mice, while treatment with eriodictyol effectively reduced the iron level in APP/PS1 mice (Fig. 4A). Meanwhile, the levels of ferrous iron and total iron in the cortex and hippocampus of mice were detected using an iron assay kit. The results are shown in Fig. 4B; compared with APP/PS1 mice, eriodictyol treatment significantly decreased the levels of ferrous iron and total iron in the cortex and hippocampus of APP/PS1 mice. Western blot analysis revealed reduced the expression of transferrin receptor (TfRC) and ferritin heavy chain (FTH) were reduced in the cortex and hippocampus of APP/PS1 mice upon eriodictyol treatment compared with that of APP/PS1 control mice. Fpn (the only iron export protein) was up-regulated in the cortex and hippocampus by eriodictyol treatment (Fig. 4C and Additional file 1: Fig. S2A). These data imply that eriodictyol treatment might maintain the iron balance in cells by reducing iron intake and increasing iron output.

MDA is a specific marker of lipid peroxidation (Jiao et al. 2015). Figure 4D shows a significantly higher MDA content in the cortex and hippocampus of APP/PS1 mice than in WT mice, while treatment with eriodictyol reversed this trend.

Finally, we investigated GPX4 expression in the brain. Western blot analysis confirmed a significantly increased in GPX4 levels in both the cortex and hippocampus of APP/PS1 mice following eriodictyol treatment (Fig. 4E and Additional file 1: Fig. S2B).

Together, these results confirmed that eriodictyol effectively inhibited ferroptosis in cells in the brains of AD model mice.

### Eriodictyol attenuated the cytotoxicity and Tau hyperphosphorylation induced by the Aβ_1–42_ oligomer in HT-22 cells

**Fig. 5:**
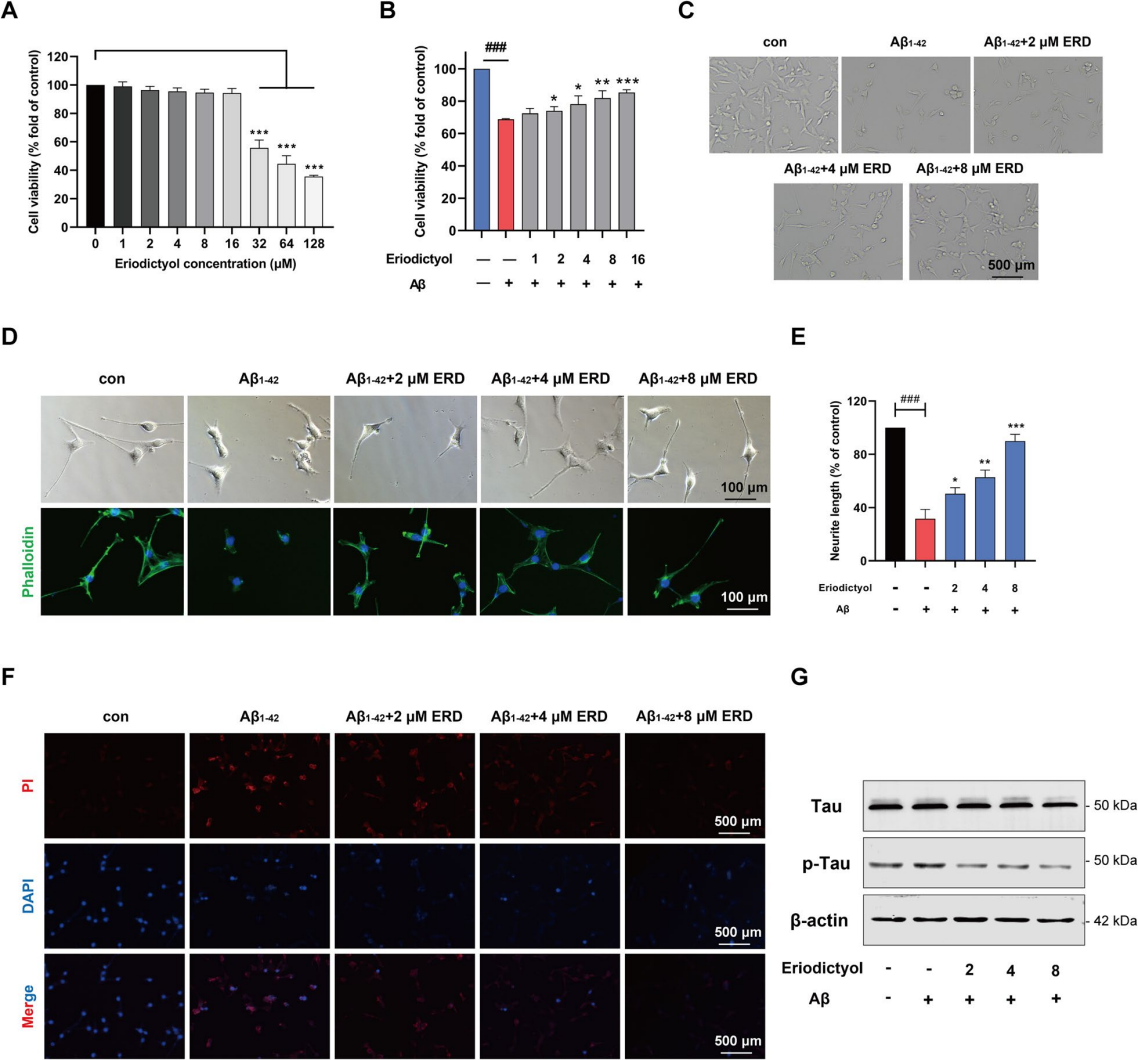
Eriodictyol attenuated the cytotoxicity and Tau hyperphosphorylation induced by the Aβ_1–42_ oligomer in HT-22 cells. **A** The viability of HT-22 cells was measured using a CCK-8 assay to explore the cytotoxicity of eriodictyol toward HT-22 cells. Cells were treated with eriodictyol (0, 2, 4, 8, 16, 32, 64 or 128 µM) for 48 h. **B** The viability of HT-22 cells was measured using a CCK-8 assay. HT-22 cells were pre-exposed to eriodictyol (0, 1, 2, 4, 8 or 16 μM) for 2 h and then treated with the Aβ_1–42_ oligomer (20 μM) for 48 h. **C** The state and number of HT-22 cells were observed under a microscope. HT-22 cells were pretreated with eriodictyol (0, 2, 4 or 8 μM) and 20 μM Aβ_1–42_ oligomer for 48 h. **D** The cytoskeleton of HT-22 cells was stained with FITC-phalloidin. HT-22 cells were treated with eriodictyol (0, 2, 4 or 8 μM) and 20 μM Aβ_1–42_ oligomer for 48 h after induction with 10 μM RA for 3 days. **E** Quantification of the neurite length of HT-22 cells using ImageJ software. **F** Cell death was detected using a propidium iodide assay. **G** Levels of Tau and p-Tau were measured using Western blotting, and β-actin served as a loading control. The data are presented as the means ± SD of three experiments. *P < 0.05, **P < 0.01 and ***P < 0.001 compared with the Aβ_1-42_ oligomer group. ^###^*P* < 0.001 compared with the control group

The HT-22 cell line was used to perform relevant experiments in vitro. First, we determined that the dosage of Aβ_1–42_ oligomer needed to construct a cell model of AD was 20 μM according to the relevant literature (Jiao et al. 2015; Ding et al. 2020). As shown in Fig. 5A, eriodictyol was not cytotoxic to HT-22 cells when administered at a concentration less than 32 μM.

We performed a series of experiments to explore whether eriodictyol relieves the cytotoxicity of the Aβ_1–42_ oligomer in HT-22 cells. The viability of HT-22 cells was markedly decreased upon Aβ_1–42_ oligomer induction, while eriodictyol treatment obviously increased cell viability in a dose-dependent manner (Fig. 5B). Meanwhile, the corresponding changes were observed in the number of surviving cells under the microscope (Fig. 5C). Then, FITC-phalloidin was applied to stain the cytoskeleton of HT-22 cells. Compared with normal HT-22 cells, the axon length of HT-22 cells treated with the Aβ_1–42_ oligomer was visibly shortened by approximately 70%, while supplementation with different dose of eriodictyol markedly reversed the effect of the Aβ_1–42_ oligomer (Fig. 5D, E). Additionally, a propidium iodide assay verified the antagonistic effect of eriodictyol on the cytotoxicity of the Aβ_1–42_ oligomer. As shown in Fig. 5F, 20 μM Aβ_1–42_ oligomer induced HT-22 cell death, while the number of dead cells was obviously reduced after treatment with different concentrations of eriodictyol. Together, these results indicated that eriodictyol treatment relieved the cytotoxicity of the Aβ_1–42_ oligomer in HT-22 cells and dramatically maintained the morphology of neuronal cells. In addition, Western blot assays were performed to determine the levels of p-Tau and Tau. Based on the results, the level of p-Tau in HT-22 cells that had been exposed to the Aβ_1–42_ oligomer was increased compared with that in the control group. Similar to the results from in vivo experiments, eriodictyol substantially inhibited hyperphosphorylation of Tau protein induced by the Aβ_1–42_ oligomer in a dose-dependent manner (Fig. 5G and Additional file 1: Fig. S3).

### Eriodictyol inhibits ferroptosis induced by the Aβ_1–42_ oligomer in HT-22 cells

**Fig. 6:**
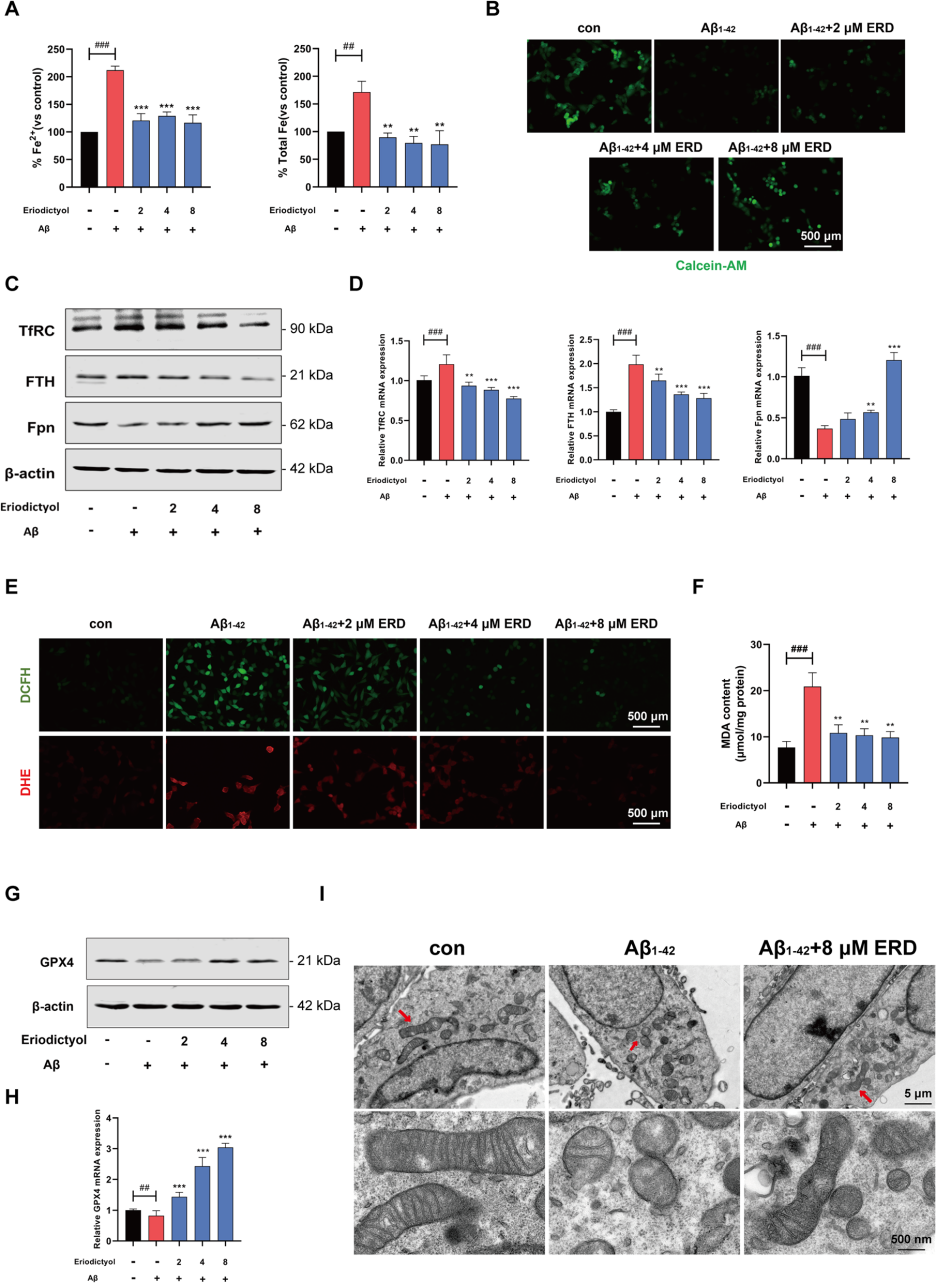
Eriodictyol inhibits ferroptosis induced by the Aβ_1–42_ oligomer in HT-22 cells. **A** The levels of ferrous iron and total iron in HT-22 cells were detected using an iron assay kit. **B** The iron content in HT-22 cells was determined using an immunofluorometric assay with calcein-AM.**C** The expression of TfRC, FTH, and Fpn was measured using Western blotting. **D** The relative mRNA expression levels of these proteins were tested using q-PCR. **E** ROS levels in HT-22 cells were measured using two types of fluorescent probes (DHE and DCFH-DA) and **F** the MDA content in HT-22 cells was measured using a Lipid Peroxidation MDA Assay Kit. **G** GPX4 protein levels in HT-22 cells were detected using Western blotting and **H** its relative mRNA expression level was measured using q-PCR. **I** The morphological features of mitochondria in HT-22 cells were observed under an electron microscope. The data are presented as the means ± SD of three experiments. **P < 0.01, and ***P < 0.001 compared with the Aβ_1–42_ oligomer group. ^##^*P* < 0.01, ^###^*P* < 0.001 compared with the control group

The levels of ferrous iron and total iron in HT-22 cells were tested using an iron assay kit. As shown in Fig. 6A, Aβ_1–42_ oligomer treatment was associated with an approximately twofold increase in the ferrous iron content and an approximately 1.5-fold increase in the total iron content. Conversely, eriodictyol noticeably reduced the increased iron aggregation induced by the Aβ_1–42_ oligomer.

Then, calcein-AM was used to stain cells to determine the iron content. Cells stained with calcein-AM emit strong green fluorescence, while metal irons quench the fluorescence. As shown in Fig. 6B, compared with the control group, the green fluorescence was dimmer in HT-22 cells treated with the Aβ_1–42_ oligomer, while eriodictyol increased the fluorescence intensity. Western blot results showed the expected changes in the expression levels of proteins related to iron metabolism (Fig. 6C and Additional file 1: Fig. S4A). Compared with Aβ_1–42_ oligomer-treated HT-22 cells, the expression levels of TfRC and FTH were decreased in HT-22 cells treated with eriodictyol in a dose-dependent manner. In contrast, the Fpn expression level in HT-22 cells was up-regulated by eriodictyol, especially at a dose of 8 μM. Moreover, the qPCR results were consistent with the Western blot assay (Fig. 6D).

Next, an immunofluorometric assay was used to detect the intracellular ROS content in HT-22 cells. Compared with the control group, the fluorescence intensity was higher in cells treated with the Aβ_1–42_ oligomer but lower in cells exposed to both the Aβ_1–42_ oligomer and eriodictyol (Fig. 6E). As expected, the MDA content in the cells was reduced by eriodictyol treatment (Fig. 6F).

Moreover, the expression of GPX4, a specific ferroptosis-related gene, was measured using Western blot and qPCR assays. Based on these results, the expression of the GPX4 protein and mRNA in HT-22 cells was down-regulated by the Aβ_1–42_ oligomer, while eriodictyol treatment up-regulated GPX4 protein and mRNA expression (Fig. 6G, H and Additional file 1: Fig. S4B).

Additionally, the morphological features of Aβ_1–42_ oligomer-treated HT-22 cells were observed under an electron microscope and included rupture and fragmentation of mitochondria with an increasing membrane density, consistent with the characteristics of ferroptosis reported in the study by Dixon (2012). Conversely, eriodictyol reversed the mitochondrial injury induced by the Aβ_1–42_ oligomer (Fig. 6I).

### Eriodictyol up-regulates VDR expression and activates the Nrf2/HO-1 signaling pathway

**Fig. 7:**
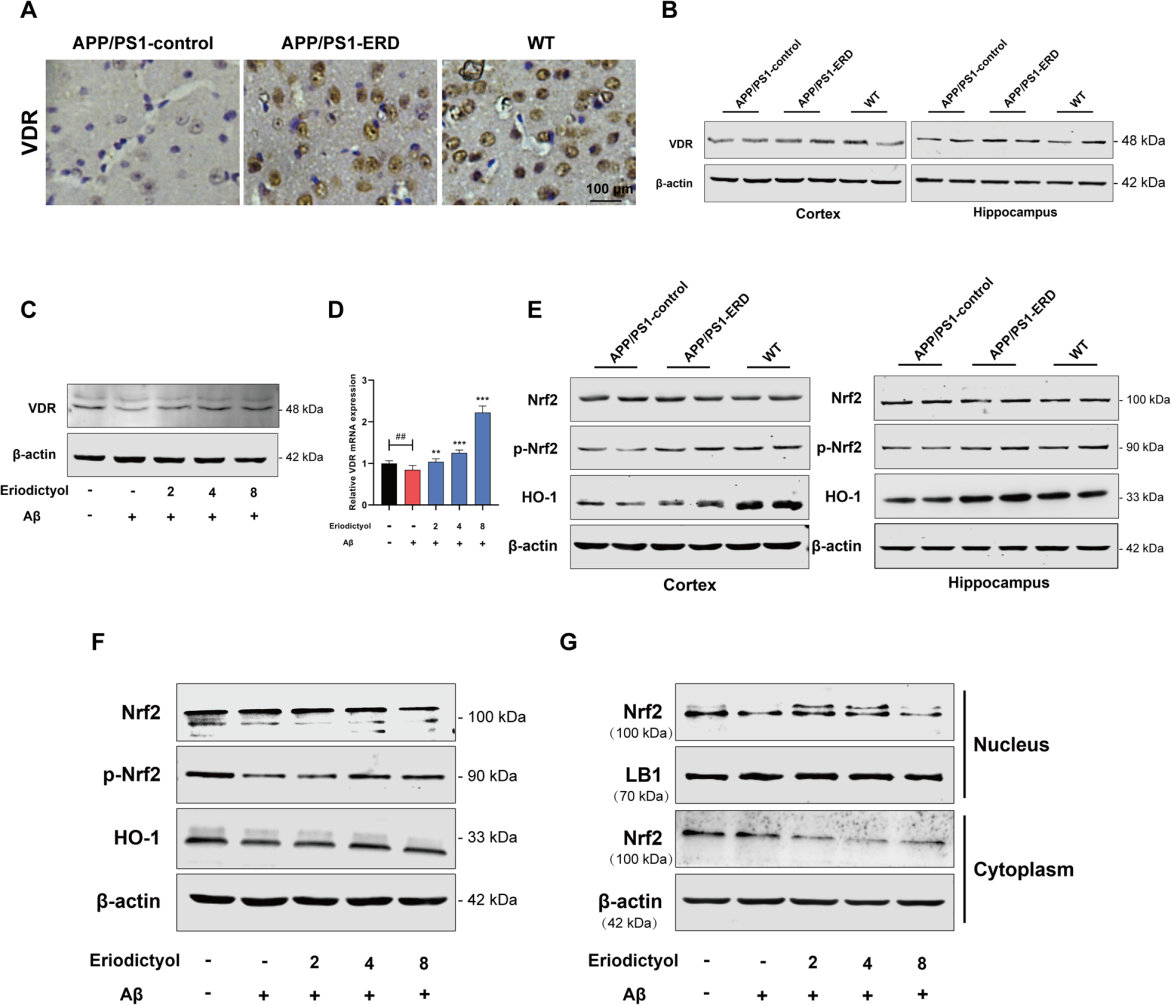
Eriodictyol up-regulates VDR expression and activates the Nrf2/HO-1 signaling pathway. **A** VDR expression in the mouse brain was detected using immunohistochemical staining. Then, **B** the VDR protein expression level in the mouse cortex and hippocampus was measured using Western blotting. **C** The VDR protein expression level in HT-22 cells was measured using Western blotting, and **D** its relative mRNA expression level was detected using q-PCR. **E** The levels of Nrf2, p-Nrf2 and HO-1 in the mouse cortex and hippocampus were measured using Western blotting. **F** The levels of Nrf2, p-Nrf2 and HO-1 in HT-22 cells were measured using Western blotting. **G** The level of Nrf2 in the nucleus and cytoplasm of HT-22 cells was detected using Western blotting, and Lamin B1 and β-actin were used as loading controls for the nuclear and cytoplasmic proteins, respectively. The data are presented as the means ± SD of three experiments. **P < 0.01 and ***P < 0.001 compared with the Aβ_1–42_ oligomer group. ^##^*P* < 0.01 compared with the control group

Some researchers discovered that nuclear factor erythroid 2-related factor 2 (Nrf2) is a key regulator of ferroptosis (Chen et al. 2017; Liu and Wang 2019). Moreover, a recent study reported that VDR was involved in ferroptosis, and VDR activation inhibited ferroptosis (Hu et al. 2020). In our study, the IHE analysis showed reduced VDR expression in APP/PS1 mice compared with WT mice. In contrast, VDR expression in APP/PS1 mice was increased upon eriodictyol treatment (Fig. 7A). The Western blot results were consistent with the IHE analysis (Fig. 7B and Additional file 1: Fig. S5A). Then, VDR expression in HT-22 cells was detected using Western blot and qPCR assays, and the result was consistent with the in vivo experiment (Fig. 7C, D and Additional file 1: Fig. S5B).

We further tested the changes in the Nrf2/HO-1 signaling pathway. As expected, the phosphorylation of Nrf2 and the expression of HO-1 were up-regulated in the cortex and hippocampus of APP/PS1 mice following eriodictyol treatment compared with the APP/PS1-control group, while total Nrf2 level not noticeably different (Fig. 7E and Additional file 1: Fig. S5C). Consistent with the in vivo results, Aβ_1–42_ oligomer treatment not only reduced the levels of phosphorylated-Nrf2 (p-Nrf2) and HO-1 but also inhibited Nrf2 entry into the nucleus. However, when HT-22 cells were treated with both the Aβ_1–42_ oligomer and eriodictyol, the levels of p-Nrf2 and HO-1 increased, and Nrf2 translocation into the nucleus was also increased (Fig. 7F, G and Additional file 1: Fig. S5D, E). These data suggested that eriodictyol increased the expression of VDR and activated the Nrf2/HO-1 signaling pathway by promoting the phosphorylation and nuclear translocation of Nrf2, but it had no effect on the total Nrf2 expression level.

### VDR is the key mediator by which eriodictyol regulates the Nrf2/HO-1 signaling pathway

**Fig. 8:**
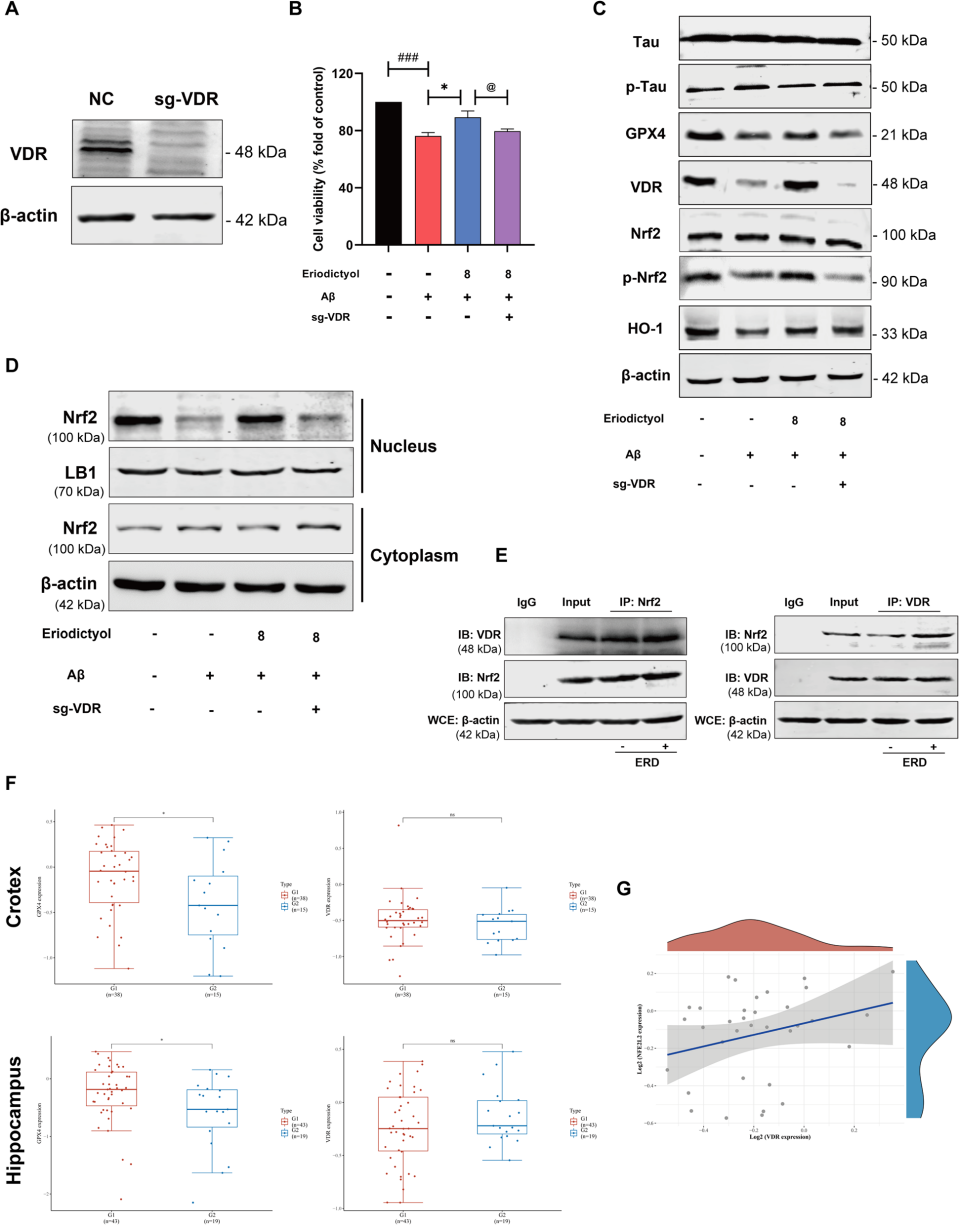
VDR is the key mediator of the regulation of the Nrf2/HO-1 signaling pathway by eriodictyol. **A** VDR was successfully knocked out by using CRISPR/CAS9 technology. **B** The viability of HT-22 cells was tested using the CCK-8 assay. Aβ_1–42_ oligomer (20 μM)-induced normal or VDR knockout HT-22 cells were treated with or without eriodictyol (8 μM) for 48 h. **C** The levels of Tau, p-Tau, GPX4, VDR, Nrf2, p-Nrf2 and HO-1 in HT-22 cells and sgVDR HT-22 cells were measured using Western blots. **D** The levels of Nrf2 in the nucleus and cytoplasm of HT-22 cells and sgVDR HT-22 cells were measured using Western blot. **E** The interaction between VDR and Nrf2 was explored by performed a coimmunoprecipitation assay. **F** The VDR and GPX4 expression levels in the cortex and hippocampus of normal elderly individuals (G1) and patients with AD (G2) were explored by performing an analysis of differentially expressed genes. **G** The relationship between Nrf2 and VDR expression in humans, R = 0.22, *P* = 0.208. The data are presented as the means ± SD of three experiments. *P < 0.05 compared with the control group. ^###^*P* < 0.001 compared with Aβ_1–42_ oligomer group. ^@^*P* < 0.05 compared with the VDR knockout group

All the results described above indicated that the VDR and Nrf2/HO-1 signaling pathways participate in the anti-ferroptosis effect of eriodictyol, but the relationship between VDR and the Nrf2/HO-1 signaling pathway is incompletely understood. As a method to further understand whether VDR plays an indispensable role in the activation of the Nrf2/HO-1 signaling pathway by eriodictyol, we used CRISPR/CAS9 technology to knock out VDR in HT-22 cells (Fig. 8A and Additional file 1: Fig. S6A). Then, sgVDR-transfected HT-22 cells and the normal group were measured using the CCK-8 assay. Eriodictyol relieved the reduction in cell viability induced by the Aβ_1–42_ oligomer, while VDR knockout reversed the protective effect of eriodictyol (Fig. 8B). In addition, we tested the expression of proteins related to AD and ferroptosis. The results are shown in Fig. 8C and Additional file 1: Fig. S6B. The phosphorylation of Tau was restrained to some extent in HT-22 cells by eriodictyol treatment compared with the Aβ_1–42_ oligomer-treated group, but the effect of eriodictyol was attenuated after VDR knockout. Meanwhile, the effect of eriodictyol on promoting GPX4 expression was reversed by VDR knockout. These results suggested that VDR knockout markedly reversed the positive effects of eriodictyol on HT-22 cells.

Subsequently, VDR knockout blocked the effect of eriodictyol on activating the Nrf2/HO-1 signaling pathway, based on the Western blot results (Fig. 8C, D and Additional file 1: Fig. S6B, C). Specifically, the levels of p-Nrf2 and HO-1 were reduced and the nuclear translocation of Nrf2 was weakened in HT-22 cells transfected with sgVDR. In addition, a Co-IP assay was conducted to further measure the interaction between VDR and Nrf2. The results confirmed that VDR interacted with Nrf2 and that eriodictyol enhanced the protein–protein interaction (Fig. 8E and Additional file 1: Fig. S6D).

Finally, a bioinformatics analysis was performed to explore the correlation between Nrf2 and VDR in patients with AD. An analysis of differentially expressed genes revealed significant differences in GPX4 expression in the cortex and hippocampus between normal elderly individuals and patients with AD, as shown in Fig. 8F. However, the expression levels of VDR did not show an obvious difference between the two groups. Next, the correlation analysis showed a positive correlation tendency between the expression of Nrf2 and VDR (Fig. 8G). Based on these results, VDR is necessary to activate the Nrf2/HO-1 signaling pathway and that eriodictyol reduces memory impairment in Alzheimer’s disease by inhibiting ferroptosis. Its mechanism is associated with the up-regulation of the Nrf2/HO-1 pathway which is mediated by VDR.

## Discussion

In recent years, the number of patients with AD has increased substantially worldwide, and Alzheimer’s disease has become an important factor affecting the quality of life of aged individuals (Patterson 2018). Unfortunately, due to the complex pathogenesis and diverse symptoms of AD, effective drugs to treat or prevent AD are currently unavailable. Eriodictyol, a natural flavonoid compound, possesses multiple biological activities, such as anti-inflammatory, antioxidant, antiradical and neuroprotective effects (Wang et al. 2017, 2020a; He et al. 2019; Jing et al. 2015). However, the mechanism of eriodictyol in AD remains ambiguous. In the present study, eriodictyol alleviated cognitive impairment in APP/PS1 mice and reduced pathological changes associated with AD both in vivo and in vitro. Mechanistically, our study implied that the anti-AD effect of eriodictyol was associated with inhibiting ferroptosis in neural cells through VDR-mediated activation of the Nrf2/HO-1 signaling pathway.

Ferroptosis is a novel form of programmed cell death, that has three main characteristics: abnormal accumulation of iron, lipid peroxidation, and a decrease in GPX4 expression (Dixon et al. 2012; Lei et al. 2019). First, intracellular iron accumulation is associated with the transport of extracellular iron and the release of intracellular iron. Specifically, the expression of transferrin (Tf), TfRC and ferritin (indicated by FTH) was increased, while the expression of Fpn, which exports iron from cells, was decreased (Dixon et al. 2012). Second, lipid peroxidation increases MDA and ROS production (Lei et al. 2019; Xie et al. 2016). Third, GPX4 is the key upstream regulator of ferroptosis (Seibt et al. 2019), and studies have shown a loss of GPX4 expression in the brain during ferroptosis, which damages biological macromolecules such as lipids and proteins (Seiler et al. 2008; Ingold et al. 2018). To date, increasing evidence supports a role for ferroptosis in AD (Masaldan et al. 2019; Weiland et al. 2019). Many studies have observed preferential iron accumulation in the cortex and hippocampus of patients with AD using magnetic resonance imaging (MRI) (Bartzokis et al. 1994, 2004; Bartzokis and Tishler 2000; Pfefferbaum et al. 2009; Bilgic et al. 2012; Langkammer et al. 2014; Ghadery et al. 2015), and iron modulates APP cleavage and Tau hyperphosphorylation (Tao et al. 2014). During iron overload, furin protein levels are reduced, which up-regulates β-secretase activity and subsequently promotes the amyloidogenesis processing of APP, causing APP depletion and Aβ deposition (Caldwell et al. 2013; Huang et al. 2017; Ward et al. 2014). Meanwhile, APP also stabilizes Fpn on the cell membrane to facilitate the efflux of iron from neurons and APP depletion leads to iron accumulation in cultured neurons and in mouse models (McCarthy et al. 2014; Wan et al. 2012; Wong et al. 2014). Additionally, GPX4 activity is of paramount importance for promoting or maintaining neuronal survival (Morris et al. 2018). This importance is graphically illustrated by an experiment showing that the ablation of GPX4 leads to the rapid degeneration of motor neurons (Chen et al. 2015). Hambright et al. also found that GPX4 knockout markedly induces degeneration of hippocampal neurons and cognitive impairment in mice (Hambright et al. 2017). Moreover, analysis of differentially expressed genes in our study showed significantly lower GPX4 expression in the cortex and hippocampus of patients with AD than that in normal elderly individuals. Therefore, ferroptosis is a potential target for the treatment or prevention of AD. In our study, we also observed the morphological and biochemical hallmarks of ferroptosis in the brains of APP/PS1 mice and Aβ_1–42_ oligomer-treated HT-22 cells while eriodictyol alleviated these changes (Figs. 4 and 6). Specifically, eriodictyol significantly reduced intracellular iron accumulation by down-regulating TfRC and FTH, up-regulating Fpn, decreasing the MDA and ROS contents, and inducing GPX4 expression. These studies verified that ferroptosis was closely related to the pathology and development of AD and suggested that eriodictyol exerts an anti-AD effect by suppressing ferroptosis.

Based on accumulating evidence, Nrf2 and its related signaling pathways play a necessary regulatory role in the process of ferroptosis (Dodson et al. 2019; Abdalkader et al. 2018). Previous reports indicated that Ser40 is located in the N-terminal Neh2 domain of Nrf2 that interacts with Keap1 in the cytoplasm. When this amino acid is phosphorylated, Nrf2 is released from Keap1, translocates into the nucleus, and then regulates ferroptosis through multiple pathways (Zeng et al. 2021). On the one hand, Nrf2 maintains cellular iron homeostasis by regulating the expression and activity of ferritin, Tf, TfRC, divalent metal-iron transporter-1 (DMT1), nuclear receptor coactivator 4 (NCOA4), Fpn, and other related proteins and regulatory factors involved in the process of iron metabolism (Dodson et al. 2019; Sun et al. 2016; Liu et al. 2020). On the other hand, Nrf2 not only regulates the expression and activity of antioxidant enzymes in the antioxidant system, such as superoxide dismutase (SOD) and HO-1 (Liu and Wang 2019; Sun et al. 2016), but also increases the expression of GPX4 by maintaining the level of glutathione in cells (Chen et al. 2017; Fan et al. 2017). Recently, researchers found that Nrf2 activation inhibits ferroptosis in cells. For instance, Zhao et al. (2021) found that Nrf2 knockdown sensitized cells to ferroptosis by down-regulating the expression of Fpn and HO-1 and then reversed the protection of autophagy inhibition in alcohol-induced HepG2 cells. The importance of Nrf2 in ferroptosis was verified again in research conducted by Li and colleagues (2021). Therefore, activating Nrf2 seems worthy of consideration to inhibit ferroptosis in cells. Additionally, a number of studies have proclaimed that the anti-AD effect of eriodictyol is related to the activation of Nrf2/HO-1 signaling pathway (Lv et al. 2019; Jing et al. 2015). In our study, we also observed that eriodictyol inhibited the pathological features of AD by activating the Nrf2/HO-1 signaling pathway, including promoting the phosphorylation (Ser40) and nuclear translocation of Nrf2, thereby up-regulating the expression of HO-1. Furthermore, VDR (a karyophilic protein, that binds 1,25(OH)_2_D_3_ to achieve its biological functions (Li et al. 1997)) was also shown to be associated with ferroptosis, and VDR activation was proposed to reverse ferroptosis-related changes in cisplatin-induced AKI through the trans-regulation of GPX4 (Hu et al. 2020). The correlation between VDR and ferroptosis was also verified in our study. Based on these results, eriodictyol induced a significant increase in VDR expression both in vivo and in vitro in a dose-dependent manner (Fig. 7). However, analysis of differential expression of VDR in patients with AD and normal elderly did not reveal apparent differences, perhaps because the number of samples was too small (Fig. 8F). In 2018, a team reported that Nrf2 transcriptional activation is promoted by VDR (Chen et al. 2019). Does eriodictyol activate the Nrf2/HO-1 signaling pathway through VDR? In our research, VDR knockout in HT-22 cells reversed the protective effect and anti-ferroptosis of eriodictyol. Then, the results of the Co-IP assay also indicated that VDR interacts with Nrf2. In addition, the bioinformatics analysis verified that VDR expression positively correlated trend with Nrf2 expression. These foundings suggested that VDR plays an indispensable role in the activation of Nrf2/HO-1 pathway by eriodictyol.

**Fig. 9:**
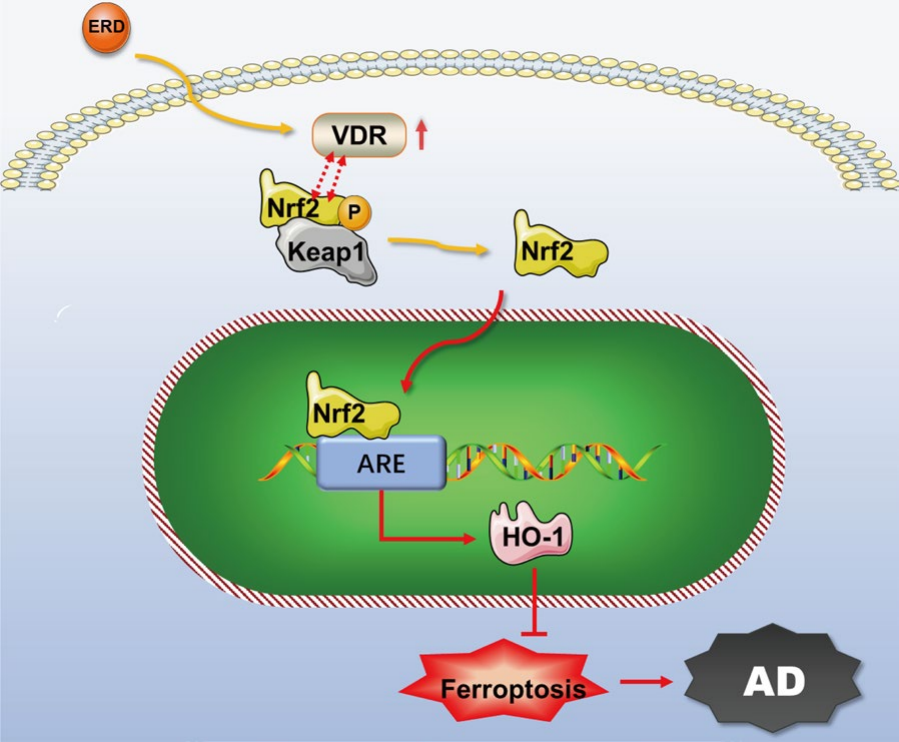
The mechanism by which eriodictyol inhibits AD. Eriodictyol alleviated cognitive injury by preventing neuronal ferroptosis, and its mechanism may be associated with VDR-mediated activation of Nrf2/HO-1 signaling. Eriodictyol up-regulated VDR expression and then activated the Nrf2/HO-1 signaling pathway by promoting the phosphorylation and nuclear translocation of Nrf2, thus regulating the expression of proteins related to ferroptosis

According to our study, we propose the following hypothesis (Fig. 9): eriodictyol alleviats cognitive injury by preventing neuronal ferroptosis, and its mechanism may be associated with VDR-mediated activation of Nrf2/HO-1 signaling pathway. Specifically, eriodictyol activates VDR, which in turn promotes Nrf2 phosphorylation (Ser40), and then Nrf2 is released from Keap1 and transported into the nucleus, thereby promoting HO-1 expression. However, this research has some limitations. We did not explore the anti-AD effect of oral administration of eriodictyol on APP/PS1 mice. We did not verify how eriodictyol up-regulates VDR in this study. These experiments will be verified in a follow-up study.

## Supplementary Information

**Additional file 1: Figure S1.** Quantification of immunoblots of Tau, p-Tau, Aβ in cortex (A) and hippocampus (B). The data are presented as the means ± SD. (*P < 0.05, **P < 0.01 and ***P < 0.001). **Figure S2.** Quantification of immunoblots of TfRC, FTH, Fpn (A) and GPX4 (B) in cortex and hippocampus. The data are presented as the means ± SD. (*P < 0.05, **P < 0.01 and ***P < 0.001). **Figure S3.** Quantification of immunoblots of Tau and p-Tau in HT-22 cells. The data are presented as the means ± SD. (**P < 0.01 and ***P < 0.001). **Figure S4.** Quantification of immunoblots of TfRC, FTH, Fpn (A) and GPX4 (B) in HT-22 cells. The data are presented as the means ± SD. (*P < 0.05, **P < 0.01 and ***P < 0.001). **Figure S5.** Quantification of immunoblots. (A, B) Quantification of immunoblots of VDR in cortex, hippocampus and HT-22 cells. (C) Quantification of immunoblots of Nrf2, p-Nrf2, HO-1 in cortex and hippocampus. (D) Quantification of immunoblots of Nrf2, p-Nrf2, HO-1 in HT-22 cells. (E) Quantification of immunoblots of Nrf2 (Nucleus/Cytoplasm) in HT-22 cells. The data are presented as the means ± SD. (*P < 0.05, **P < 0.01 and ***P < 0.001). **Figure S6.** Quantification of immunoblots. (A) Quantification of the VDR expression. (B) Quantification of immunoblots of Tau, p-Tau, GPX4, VDR, Nrf2, p-Nrf2, HO-1 in VDR knockout cells. (C) Quantification of immunoblots of Nrf2 (Nucleus/Cytoplasm) in VDR knockout cells. (D) Quantification of immunoblots of Co-IP. The data are presented as the means ± SD. (*P < 0.05, **P < 0.01, ***P < 0.001, ^##^*P* < 0.01, ^###^*P* < 0.001, ^@^*P* < 0.05, ^@@^*P* < 0.01, ^@@@^*P* < 0.001).

## Data Availability

The datasets used and/or analysed during the current study are available from the corresponding author on reasonable request.

## Abbreviations

AD: Alzheimer disease
ERD: Eriodictyol
APP/PS1 mice: APPswe/PS1E9 transgenic mice
WT mice: Wild-type C57 mice
i.p.: Intraperitoneal injection
HT-22 cell: Mouse hippocampal neuron cell line
Aβ: Amyloid-β
Nrf2: Nuclear factor erythroid 2-related factor 2
HMOX1 or HO-1: Heme oxygenase-1
VDR: Vitamin D receptor
p-Tau: Phosphorylated-Tau
TfRC: Transferrin receptor
FTH: Ferritin heavy chain
p-Nrf2: Phosphorylated-Nrf2
Fpn: Ferroportin-1
GPX4: Glutathione peroxidase type 4
ROS: Reactive oxygen species
MDA: Malondialdehyde
DHE: Dihydroethidium
DCFH-DA: Dihydrodichlorofluorescein diacetate
