# Supplementary material for: Eriodictyol ameliorates cognitive dysfunction in APP/PS1 mice by inhibiting ferroptosis via vitamin D receptor-mediated Nrf2 activation
Source: Mol Med. 2022 Jan 29;28:11. doi: 10.1186/s10020-022-00442-3 (PMC8800262; doi:10.1186/s10020-022-00442-3)
Supplement: Supplementary file 1 — Additional file 1: Figure S1. Quantification of immunoblots of Tau, p-Tau, Aβ in cortex (A) and hippocampus (B). The data are presented as the means ± SD. (*P < 0.05, **P < 0.01 and ***P < 0.001). Figure S2. Quantification of immunoblots of TfRC, FTH, Fpn (A) and GPX4 (B) in cortex and hippocampus. The data are presented as the means ± SD. (*P < 0.05, **P < 0.01 and ***P < 0.001). Figure S3. Quantification of immunoblots of Tau and p-Tau in HT-22 cells. The data are presented as the means ± SD. (**P < 0.01 and ***P < 0.001). Figure S4. Quantification of immunoblots of TfRC, FTH, Fpn (A) and GPX4 (B) in HT-22 cells. The data are presented as the means ± SD. (*P < 0.05, **P < 0.01 and ***P < 0.001). Figure S5. Quantification of immunoblots. (A, B) Quantification of immunoblots of VDR in cortex, hippocampus and HT-22 cells. (C) Quantification of immunoblots of Nrf2, p-Nrf2, HO-1 in cortex and hippocampus. (D) Quantification of immunoblots of Nrf2, p-Nrf2, HO-1 in HT-22 cells. (E) Quantification of immunoblots of Nrf2 (Nucleus/Cytoplasm) in HT-22 cells. The data are presented as the means ± SD. (*P < 0.05, **P < 0.01 and ***P < 0.001). Figure S6. Quantification of immunoblots. (A) Quantification of the VDR expression. (B) Quantification of immunoblots of Tau, p-Tau, GPX4, VDR, Nrf2, p-Nrf2, HO-1 in VDR knockout cells. (C) Quantification of immunoblots of Nrf2 (Nucleus/Cytoplasm) in VDR knockout cells. (D) Quantification of immunoblots of Co-IP. The data are presented as the means ± SD. (*P < 0.05, **P < 0.01, ***P < 0.001, ##P < 0.01, ###P < 0.001, @P < 0.05, @@P < 0.01, @@@P < 0.001). [file 10020_2022_442_MOESM1_ESM.docx]

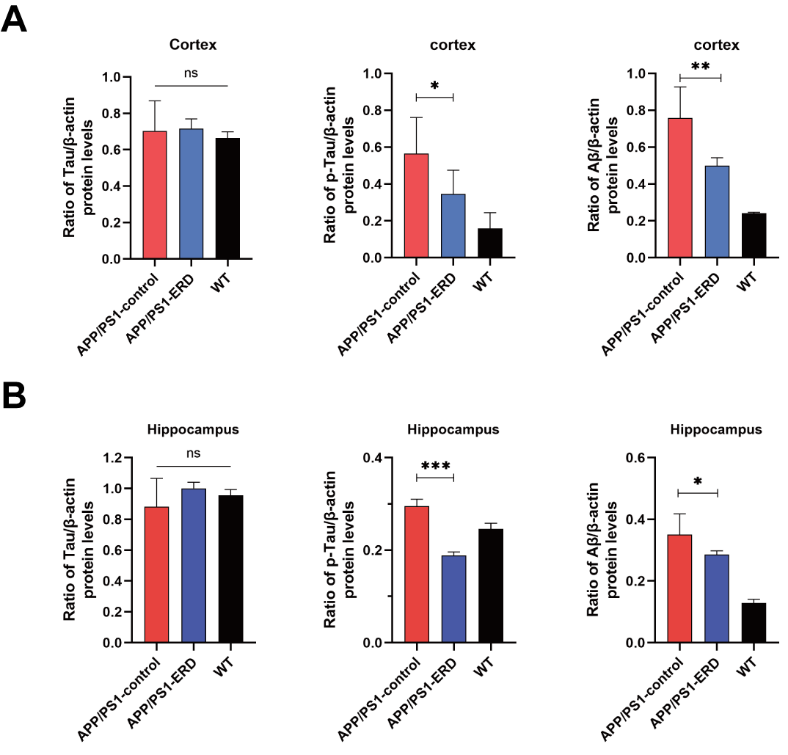


**Figure S1** Quantification of immunoblots of Tau, p-Tau, Aβ in cortex (**A**) and hippocampus (**B**). The data are presented as the means ± SD. (**P* < 0.05, ** *P* < 0.01 and ****P* < 0.001)


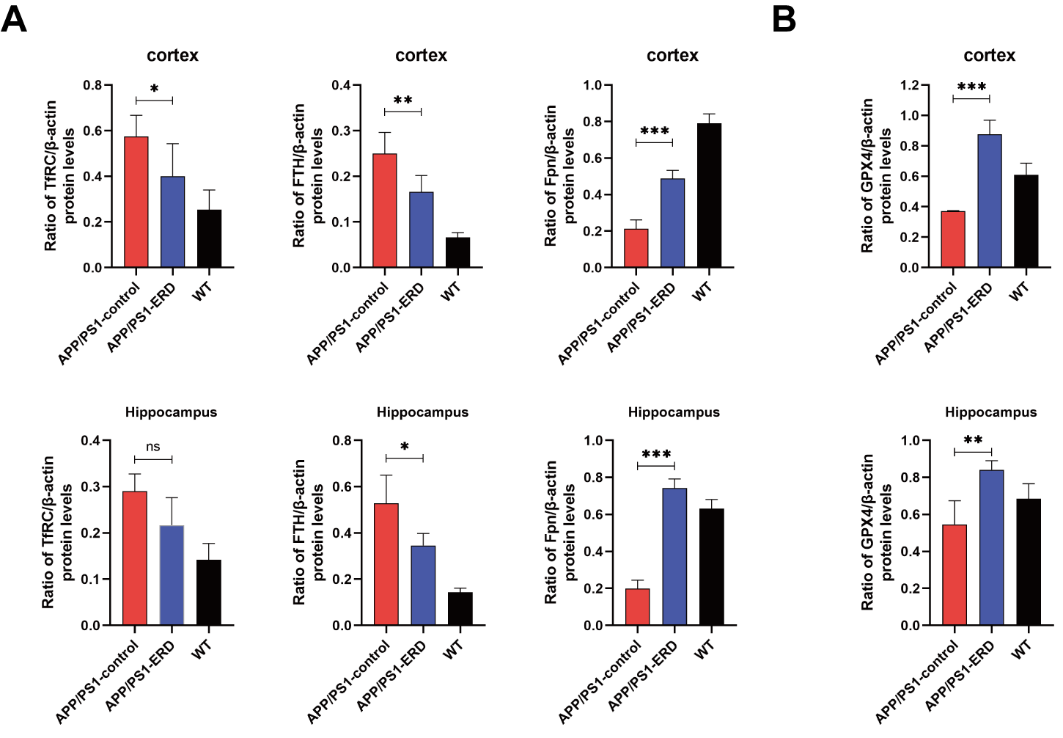


**Figure S2** Quantification of immunoblots of TfRC, FTH, Fpn (**A**) and GPX4 (**B**) in cortex and hippocampus. The data are presented as the means ± SD. (**P* < 0.05, ** *P* < 0.01 and ****P* < 0.001)


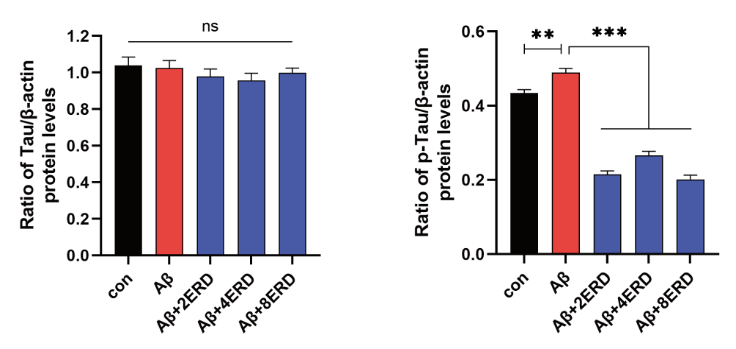


**Figure S3** Quantification of immunoblots of Tau and p-Tau in HT-22 cells. The data are presented as the means ± SD. (** *P* < 0.01 and ****P* < 0.001)


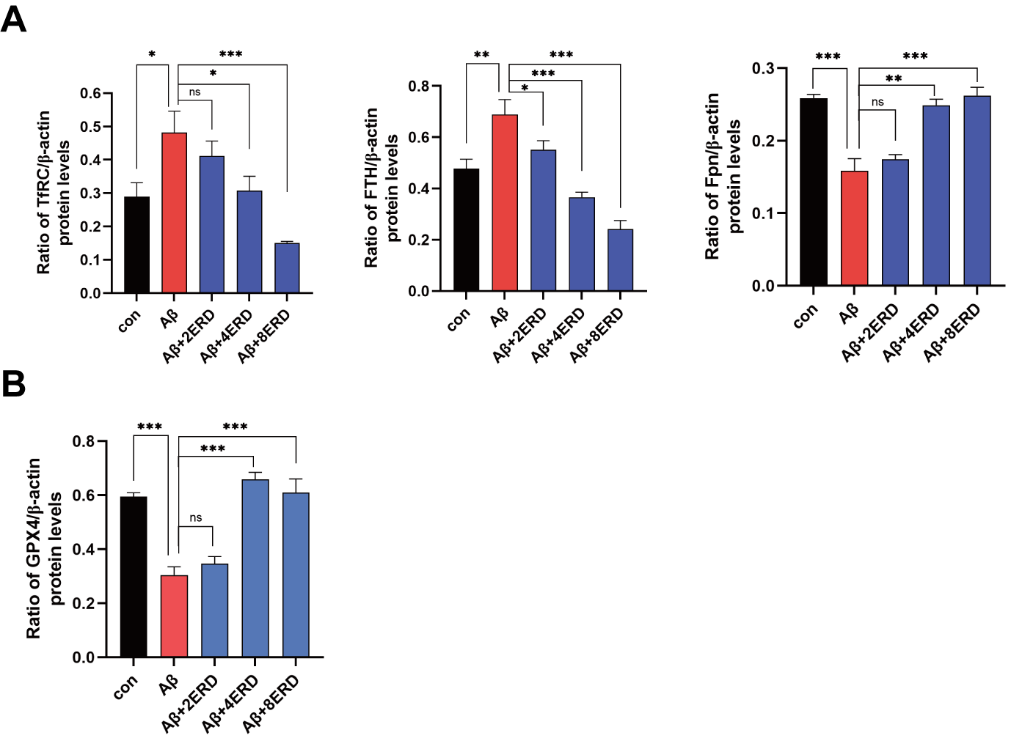


**Figure S4** Quantification of immunoblots of TfRC, FTH, Fpn (**A**) and GPX4 (**B**) in HT-22 cells. The data are presented as the means ± SD. (**P* < 0.05, ** *P* < 0.01 and ****P* < 0.001)


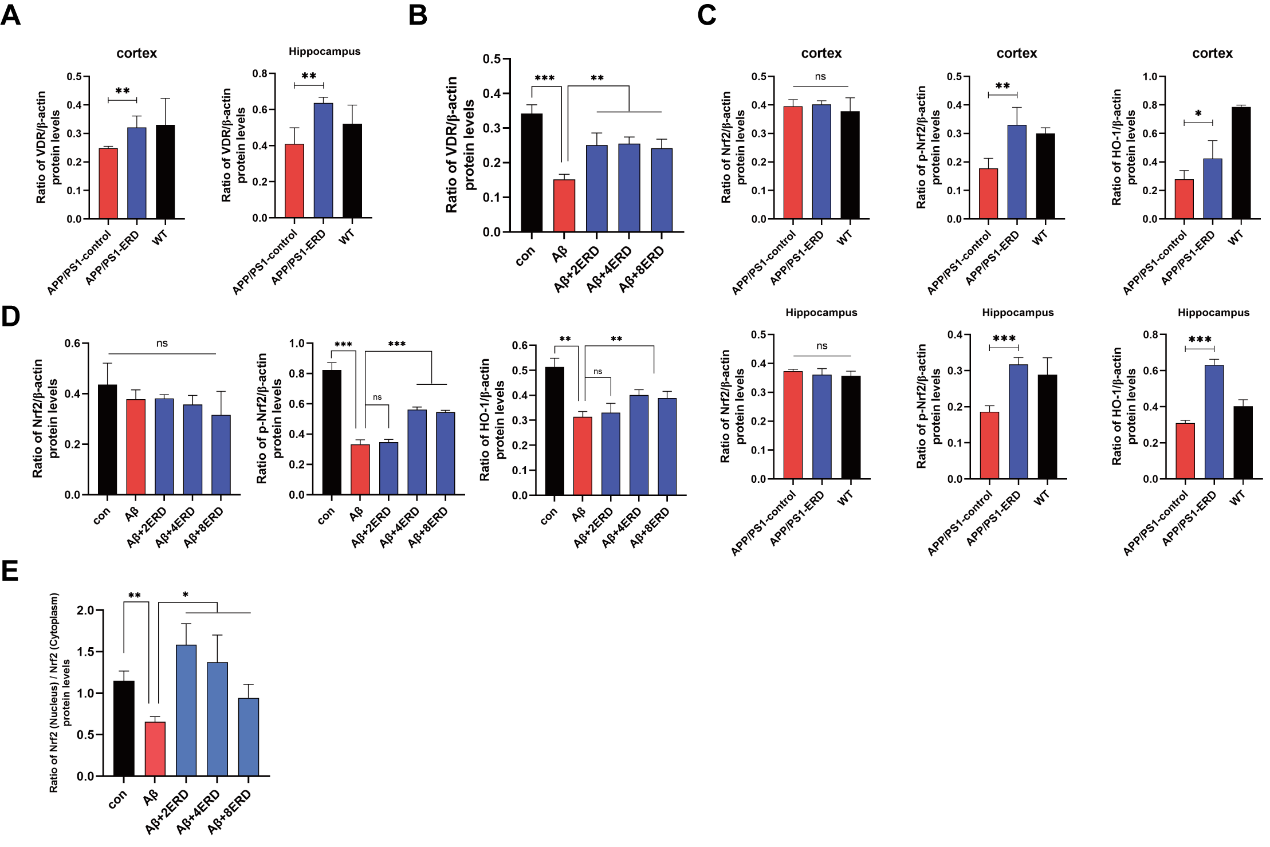


**Figure S5** Quantification of immunoblots. (**A-B**) Quantification of immunoblots of VDR in cortex, hippocampus and HT-22 cells. (**C**) Quantification of immunoblots of Nrf2, p-Nrf2, HO-1 in cortex and hippocampus. (**D**) Quantification of immunoblots of Nrf2, p-Nrf2, HO-1 in HT-22 cells. (**E**) Quantification of immunoblots of Nrf2 (Nucleus/Cytoplasm) in HT-22 cells. The data are presented as the means ± SD. (**P* < 0.05, ** *P* < 0.01 and ****P* < 0.001)


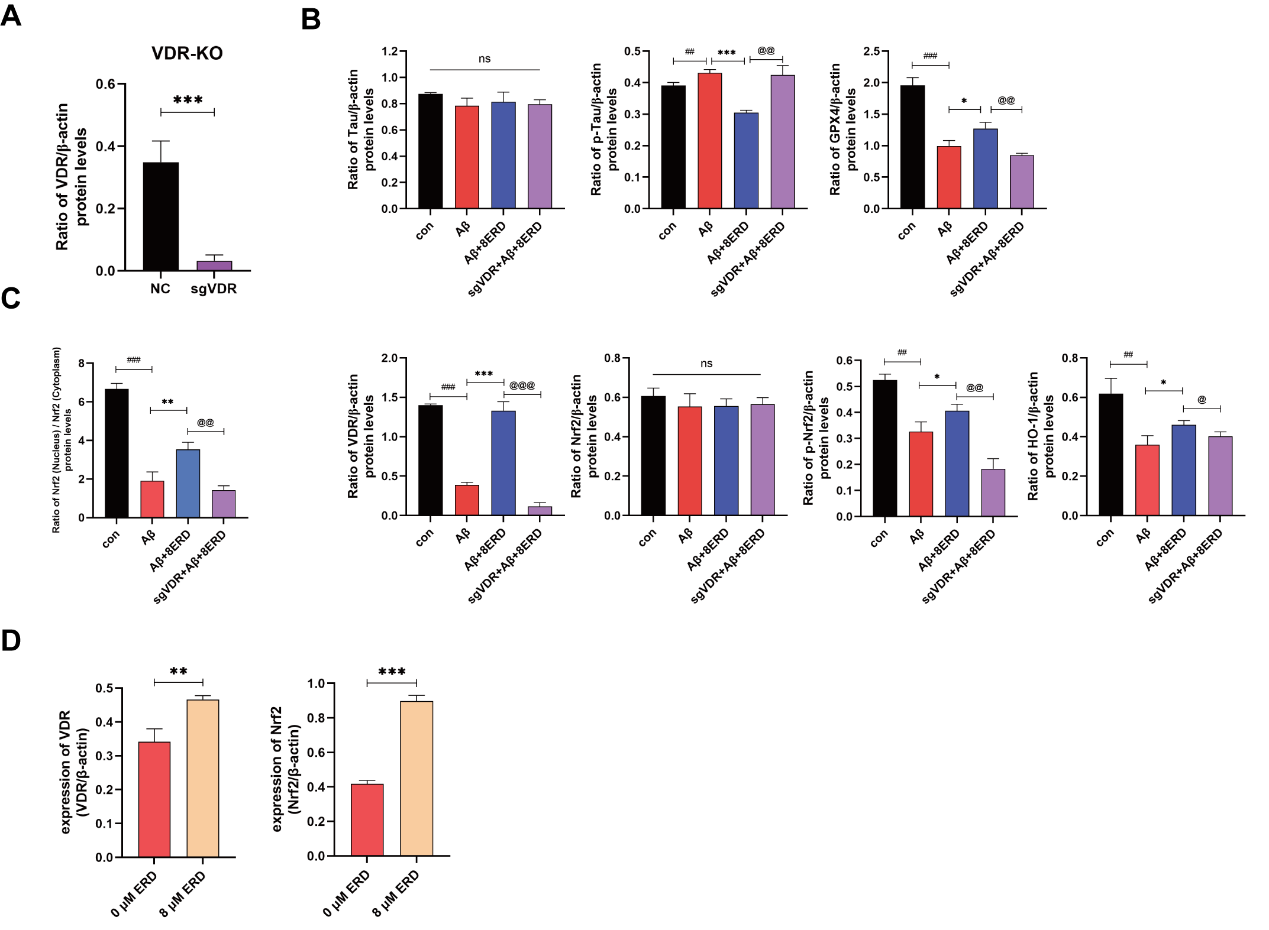


**Figure S6** Quantification of immunoblots. (**A**) Quantification of the VDR expression. (**B**) Quantification of immunoblots of Tau, p-Tau, GPX4, VDR, Nrf2, p-Nrf2, HO-1 in VDR knockout cells. (**C**) Quantification of immunoblots of Nrf2 (Nucleus/Cytoplasm) in VDR knockout cells. (**D**) Quantification of immunoblots of Co-IP. The data are presented as the means ± SD. (**P* < 0.05, ** *P* < 0.01, ****P* < 0.001, ## *P* < 0.01, ### *P* < 0.001, @ *P* < 0.05, @@ *P* < 0.01, @@@*P* < 0.001)
